# Community Structure Analysis of Transcriptional Networks Reveals Distinct Molecular Pathways for Early- and Late-Onset Temporal Lobe Epilepsy with Childhood Febrile Seizures

**DOI:** 10.1371/journal.pone.0128174

**Published:** 2015-05-26

**Authors:** Carlos Alberto Moreira-Filho, Silvia Yumi Bando, Fernanda Bernardi Bertonha, Priscila Iamashita, Filipi Nascimento Silva, Luciano da Fontoura Costa, Alexandre Valotta Silva, Luiz Henrique Martins Castro, Hung-Tzu Wen

**Affiliations:** 1 Department of Pediatrics, Faculdade de Medicina da Universidade de São Paulo (FMUSP), São Paulo, SP, Brazil; 2 Instituto de Física de São Carlos, Universidade de São Paulo, São Carlos, SP, Brazil; 3 Graduate Program on Experimental Pathophysiology, FMUSP, São Paulo, SP, Brazil; 4 Department of Neurology, FMUSP, São Paulo, SP, Brazil; 5 Clinical Neurology Division, Hospital das Clínicas, FMUSP, São Paulo, SP, Brazil; 6 Epilepsy Surgery Group, Hospital das Clínicas, FMUSP, São Paulo, SP, Brazil; Royal College of Surgeons in Ireland, IRELAND

## Abstract

Age at epilepsy onset has a broad impact on brain plasticity and epilepsy pathomechanisms. Prolonged febrile seizures in early childhood (FS) constitute an initial precipitating insult (IPI) commonly associated with mesial temporal lobe epilepsy (MTLE). FS-MTLE patients may have early disease onset, i.e. just after the IPI, in early childhood, or late-onset, ranging from mid-adolescence to early adult life. The mechanisms governing early (E) or late (L) disease onset are largely unknown. In order to unveil the molecular pathways underlying E and L subtypes of FS-MTLE we investigated global gene expression in hippocampal CA3 explants of FS-MTLE patients submitted to hippocampectomy. Gene coexpression networks (GCNs) were obtained for the E and L patient groups. A network-based approach for GCN analysis was employed allowing: i) the visualization and analysis of differentially expressed (DE) and complete (CO) - all valid GO annotated transcripts - GCNs for the E and L groups; ii) the study of interactions between all the system’s constituents based on community detection and coarse-grained community structure methods. We found that the E-DE communities with strongest connection weights harbor highly connected genes mainly related to neural excitability and febrile seizures, whereas in L-DE communities these genes are not only involved in network excitability but also playing roles in other epilepsy-related processes. Inversely, in E-CO the strongly connected communities are related to compensatory pathways (seizure inhibition, neuronal survival and responses to stress conditions) while in L-CO these communities harbor several genes related to pro-epileptic effects, seizure-related mechanisms and vulnerability to epilepsy. These results fit the concept, based on fMRI and behavioral studies, that early onset epilepsies, although impacting more severely the hippocampus, are associated to compensatory mechanisms, while in late MTLE development the brain is less able to generate adaptive mechanisms, what has implications for epilepsy management and drug discovery.

## Introduction

Mesial temporal lobe epilepsy with childhood febrile seizures (FS-MTLE) is a distinctive entity that can be delineated from afebrile MTLE as demonstrated by epidemiological [1], radiological [2], and genomic [3, 4] studies. The age at onset in FS-MTLE is trimodal, with peaks at early childhood, adolescence, and early adult-life [5]. The age of seizure onset exerts a relevant impact on brain activity and connectivity because epilepsy-associated processes interfere with normal brain developmental changes, as evidenced by fMRI and network (graph theory) computational studies of brain connectivity [6, 7, 8]. These studies show that late-onset MTLE causes more pronounced neuronal network alterations (whole-brain properties), since the mature brain has a diminished capacity to generate adaptive responses to epilepsy effects, whereas in early-onset MTLE several compensatory mechanisms are activated in the more plastic younger brain. On the other hand, early MTLE onset is associated to a more severe functional abnormality in the ictal hippocampus (local alteration) [8], what is in agreement with the inverse correlation between age of seizure onset and severity of mesial temporal sclerosis [9]. About 40% of the patients with FS-MTLE develop refractory epilepsy [10] and early onset of seizures is a predictive factor for pharmacoresistancy [11, 12].

Prolonged febrile seizures (FS) and febrile status epilepticus (FSE) in early childhood have long been associated to a higher risk of temporal lobe epilepsy and mesial temporal sclerosis, but a causal relationship was just recently established, based on epidemiological and imaging investigations, as well as on studies with animal models (reviewed in [13]). FS and FSE can cause hippocampal injury due to the interplay between inflammation and fever: fever increases neuronal firing and causes the overexpression of inflammatory molecules (IL-1β, TNF-α, HMGB1), leading to neuronal injury, neuronal excitability and epileptogenesis [14, 15, 16]. Studies in animal models showed that a single episode of neonatal seizure permanently alters glutamatergic synapses [17]. In fact, initial precipitating injuries, such as complex febrile seizures, are potent inducers of epigenetic alterations that modify brain functioning [18]. It was shown that DNA methylation is an early event triggered by FSE that may persists late in the epileptic hippocampus, leading to permanent changes in gene expression [19].

The predisposition to developing temporal lobe epilepsy and hippocampal sclerosis has been investigated in animal models of FS induced by hyperthermia and in prospective clinical studies of children with FSE. Altogether, these studies revealed that FS development and subsequent epilepsy results from a combination of environmental and genetic factors that vary in each individual [13, 20, 21]. In rodent models of FS-like seizures induced by hyperthermia, a quite regular latency period between the initial insult and the development of recurrent seizures is always observed [22], but in human FS-MTLE the age at onset varies from early childhood to adult life [5, 1]. The mechanisms governing early (E) or late (L) disease onset in FS-MTLE are largely unknown but their unraveling is crucial, since the latent period could be a therapeutic window for developing antiepileptogenic drugs [23].

In order to look into the molecular mechanisms leading to early or late FS-MTLE onset, we have decided to investigate comparatively the hippocampal CA3 transcriptional profile of a group of FS-MTLE patients where all individuals had their IPI before 4 years of age but who developed MTLE in early childhood or in mid-adolescence and adult life. Our rationale was based on the evidences that FS-induced epigenetic changes produce lasting effects on gene expression in human hippocampus, and in the tenets of network medicine: i) genes do not operate in isolation but as components of complex networks [24, 25, 26]; ii) genomic interaction data is basically composed of pairwise relationships among transcriptional modules (network communities [27]); iii) complex diseases rarely derive from alterations in a single gene but, on the contrary, reflect perturbations in cell’s genomic and protein-protein interaction networks, often caused by environmental factors [28, 29, 30]. Therefore, we sought to find out how FS, an initial precipitating insult of environmental origin, differentially impacted hippocampal CA3 gene coexpression networks of FS-MTLE patients with early or late disease onset. Conceivably, different precipitating insult effects on disease onset could arise from pre-existent CA3 gene network differences between E and L patients, due to inherited genetic differences, or stem from different network adaptations to the insult, also influenced by allelic differences among individuals. In order to investigate this issue, gene expression data was analyzed using network science parameters, i.e. with emphasis in complex network visualization, gene hierarchy categorization, community detection and coarse-grained community structure [4, 26, 31, 32].

## Material and Methods

### Patients

#### Ethics Statement

The patients with refractory MTLE and febrile IPI included in this study were selected through the CInAPCe-FAPESP Program (www.fapesp.br/en/; www.cinapce.org.br). This research has been approved by the research ethics committees of Hospital das Clínicas da FMUSP and of Hospital Albert Einstein under numbers 251/05 and CAEE 0122.0.028.174.05 respectively. A written informed consent was obtained from all patients.

Refractory epilepsy cases were defined as those who have not gained seizure control after treatment with three or more anticonvulsant drugs. In the last 3–4 years before surgery, seizure control was attempted with carbamazepine, oxicarbazepine, phenobarbital, clobazam, topiramate, and lamotrigine, in different drug combinations. The patients were submitted to clinical, electrophysiological, neuropsychological and neuroimaging evaluations before surgery. All patients included in this study (Table 1) had prolonged febrile seizures as the IPI at or before the age of 4 years. Early onset patients were those who developed the disease soon after the IPI, whereas the late onset patients developed the disease after ≥13 years old. In the present investigation we compared global gene expression profiles of CA3 explants obtained at surgery room from seven early-onset (group E) and seven late-onset RMTLE patients (group L) submitted to corticoamigdalohippocampectomy. E and L groups had the same gender composition: three males and four females. Hippocampal hypersignal was observed in T2-weighted MRimages in all cases, what is a hallmark of hippocampal sclerosis [33]. MRI evidence indicated that six of the E patients had MTLE on the right side and one on the left side, whereas five of the L patients had MTLE on the left side and two on the right side. Patients with bilateral hippocampal sclerosis, lesions other than hippocampal sclerosis (tumors, dysplasias, etc), and psychiatric disorders were not included in this study. No significant group differences were found for epilepsy duration or age at surgery between E and L groups (S1 Fig). All surgical specimens were classified as ILAE type 1 hippocampal sclerosis [34], that is, severe neuronal cell loss and gliosis predominantly in CA1 and CA4 regions.

**Table 1:** Patients' clinical and demographic data.

			Epilepsy				
Patient ID	Gender	FR	IPI (yr/mo)	Onset (yr/mo)	Duration (yr/mo)	Age at surgery (yr)	Side
E1	M	No	4yr	4yr	9yr	13	R
E2	M	No	4yr	4yr	35yr	39	L
E3	M	2^nd^	2yr	2yr	31yr	33	R
E4	F	No	6mo	6mo	55yr6mo	56	R
E5	F	1^st^/3^rd^	8mo	9mo	28yr3mo	29	R
E6	F	No	2yr	2yr	18yr	20	R
E7	F	1^st^/3^rd^	3yr	5yr	42yr	47	R
L1	M	2^nd^	3yr	14yr	9yr	23	L
L2	F	2^nd^	2yr	29yr	13yr	42	L
L3	M	2^nd^	6mo	15yr	14yr	29	L
L4	F	No	2yr	14yr	14yr	28	R
L5	M	2^nd^	2yr	19yr	31yr	50	L
L6	F	No	9mo	13yr	11yr	24	R
L7	F	1^st^	6mo	16yr	38yr	54	L

E-Early onset; L-Late onset; FR-Familial recurrence; IPI-Initial precipitant insult; 1^st^/2^nd^/3^rd^- first, second or third degree relative with epilepsy.

### Brain tissue specimens for gene expression and neuropathological studies

Fresh ex-vivo explants from hippocampal CA3 of our patients were obtained at the surgery room and immediately preserved with RNAlater (Qiagen cat. no. 76106, Valencia, CA). MRI and histological studies were performed in all removed hippocampi for neuropathology analysis and for confirming that the explants for genomic studies were obtained at the proper site [2, 3].

### RNA extraction

Brain tissue explants from CA3 (3–4 mm3) were homogenized with TissueRupter (Qiagen, cat. no. 9001272 Valencia, CA) and total RNA was extracted from the homogenates using the RNeasy Lipid Tissue Kit (Qiagen cat. no. 74804, Valencia, CA) according to the manufacturer’s instructions. RNA quality was assessed on the Agilent BioAnalyzer 2100 (Agilent, Santa Clara, CA). All samples were stored at -80°C until used in hybridization experiments.

### Microarray hybridization and gene expression analysis

In order to determine gene expression profiles, 4x44K DNA microarrrays (Whole Human Genome Microarray Kit, Agilent Technologies, cat no. G4112F, Santa Clara, CA) were used. The procedures for hybridization followed the protocols provided by the manufacturer´s instructions (One-Color Microarray-Based Gene Expression Analysis—Quick Amp Labeling). The images were captured by the reader Agilent Bundle according to the parameters recommended for bioarrays and extracted by Agilent Feature Extraction software version 9.5.3 and considering spots present none or only one flag (i.e. low intensity, saturation, controls, etc.). The selected transcripts were used for analysis using the R software version 2.11.1 (R Development Core Team, 2010). We identified 13,427 valid GO annotated genes for the CA3 samples (early- and late-onset patients). By means of the TMEV software version 4.6.1 we obtained the differentially expressed (DE) Gene Ontology (GO) annotated genes using the t-test (P<0.05). All microarray raw data has been deposited in GEO public database (http://www.ncbi.nlm.nih.gov/geo), a MIAME compliant database, under accession number GSE57585. Differential gene expression data were validated through quantitative real-time polymerase chain reaction [3].

### Gene coexpression networks (GCNs): visualization, analysis and community detection

Gene coexpression networks for differentially expressed GO annotated genes (DE) and for all valid GO annotated genes (CO) were constructed for E and L groups based on Pearson’s correlation, as we previously described [4]. Pearson’s correlation identifies sets of genes which covaries (positively or negatively), thus allowing us to construct networks by considering nodes as genes, with edges inferred if a pair presents high absolute value of correlation. Specifically, we define a correlation threshold that determines if edges are present or absent in the resulting network. This is done in a way that all nodes are connected to the major component and the network is stable in the sense that slight changes in the threshold value do not significantly affect its topological structure [4]. Networks were tested for scale free status by Kolmogorov-Smirnov (K-S) statistics, i.e. power law distributions in empirical data [35].

As these networks may grow larger in the number of components (e.g. tens of thousands genes) or present very intricate connections between them (such as hierarchical or modular structure), it becomes mandatory the use of tools and methodologies borrowed from network science to better characterize such systems.

We developed a network methodology for GCN visualization (3D) and analysis [4] that allows the categorization of network nodes according to node-centered connectivity taken along distinct hierarchical levels of gene-gene neighborhoods [36, 37]: hubs are highly connected nodes, VIPs—standing for “Very Important Person”, an acronym initially coined for the study of social networks [38] and equivalent to the term “date-hubs” in biological network papers [39]—have low node degree but connect only with hubs, and high-hubs have VIP status and high overall number of connections. We classified network nodes as VIPs, hubs or high-hubs by obtaining the node degree, *k*_*0*_, and the first level concentric node degree, *k*_*1*_, which takes into account all node connections leaving from its immediate neighborhood, then projecting all node values in a *k*_*0*_ vs *k*_*1*_ graphic. All calculations were done by using Python program and the conceptual framework is described at http://cyvision.if.sc.usp.br/~bant/hierarchical/.

#### Connectivity

The network connectivity k for non-directed networks was calculated by k = 2L/N, where L stands for the number of edges and N for the number of nodes [40].

#### Community detection

Community detection in complex networks is usually accomplished by discovering the network modular structure that optimizes the modularity measurement. Modularity takes into account the relationship between the number of links inside a community and between nodes in distinct communities compared to the random model [27; 40]. A diverse range of optimization techniques exist to optimize the modularity. Here we applied the method proposed by Blondel et al. [41] which attains good modularity values and presents excellent performance.

#### Coarse-grained community structure

As a complementary analysis for the community detection, each GCN was rearranged in a new network accounting only for the relationships between each community, also known as coarse-grained community structure (CGCS) [42]. Here the CGCS was generated by contracting all nodes inside each community into a single community node, likewise, edges are added up as connection weights between such communities. This structure can also be obtained directly by considering the mixing matrix [27] as an adjacency matrix of the new network.

### Interactome analysis

The interactome networks were constructed using an in house free web tool developed by Leandro de A. Lima and Renato D. Puga—Centro Internacional de Pesquisa e Ensino (CIPE)—Hospital A. C. Camargo (http://bioinfo.lbhc.hcancer.org.br/cgi-bin/interactomegraph/index.cgi). Only genes categorized as hubs, VIPs or high hubs were considered in this analysis. MINT and IntAct databases (experimentally verified protein-protein interactions) were selected for comparison and data generation. Data analysis and visualization were accomplished through Cytoscape (version 3.1.0, www.cytoscape.org).

## Results

### GCN analyses

**Fig 1:**
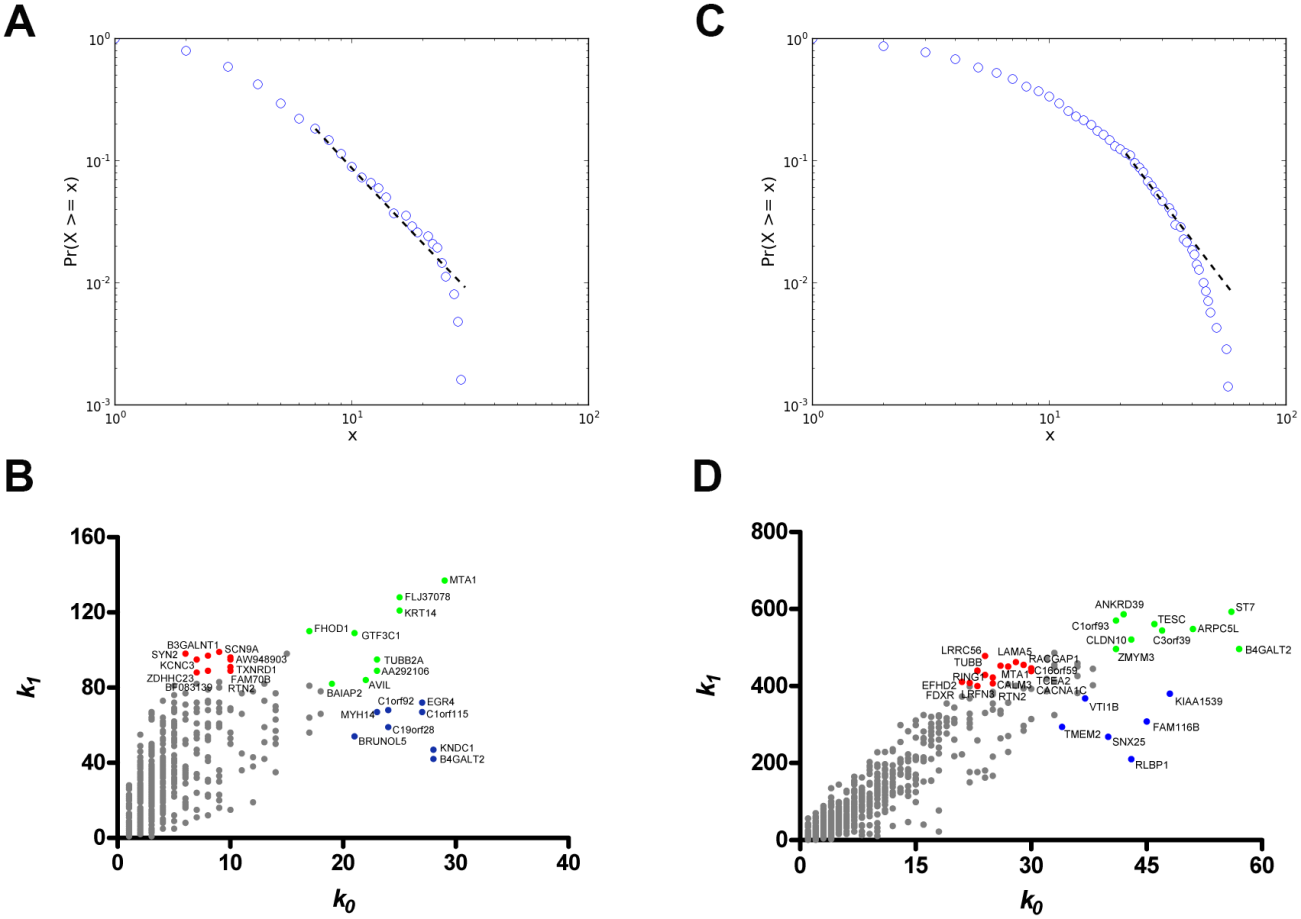
Node distribution and categorization for DE networks. Kolmogorov-Smirnov test for scale free status for E-DE (A) and L-DE (B) gene coexpression networks (GCNs). Scatter plots of node degree (k0) vs concentric node degree (k1) measures of GO annotated genes in E-DE (C) and L-DE networks (D). Hubs (blue), VIPs (red) and high-hubs (green), identified by their gene symbols.

**Fig 2:**
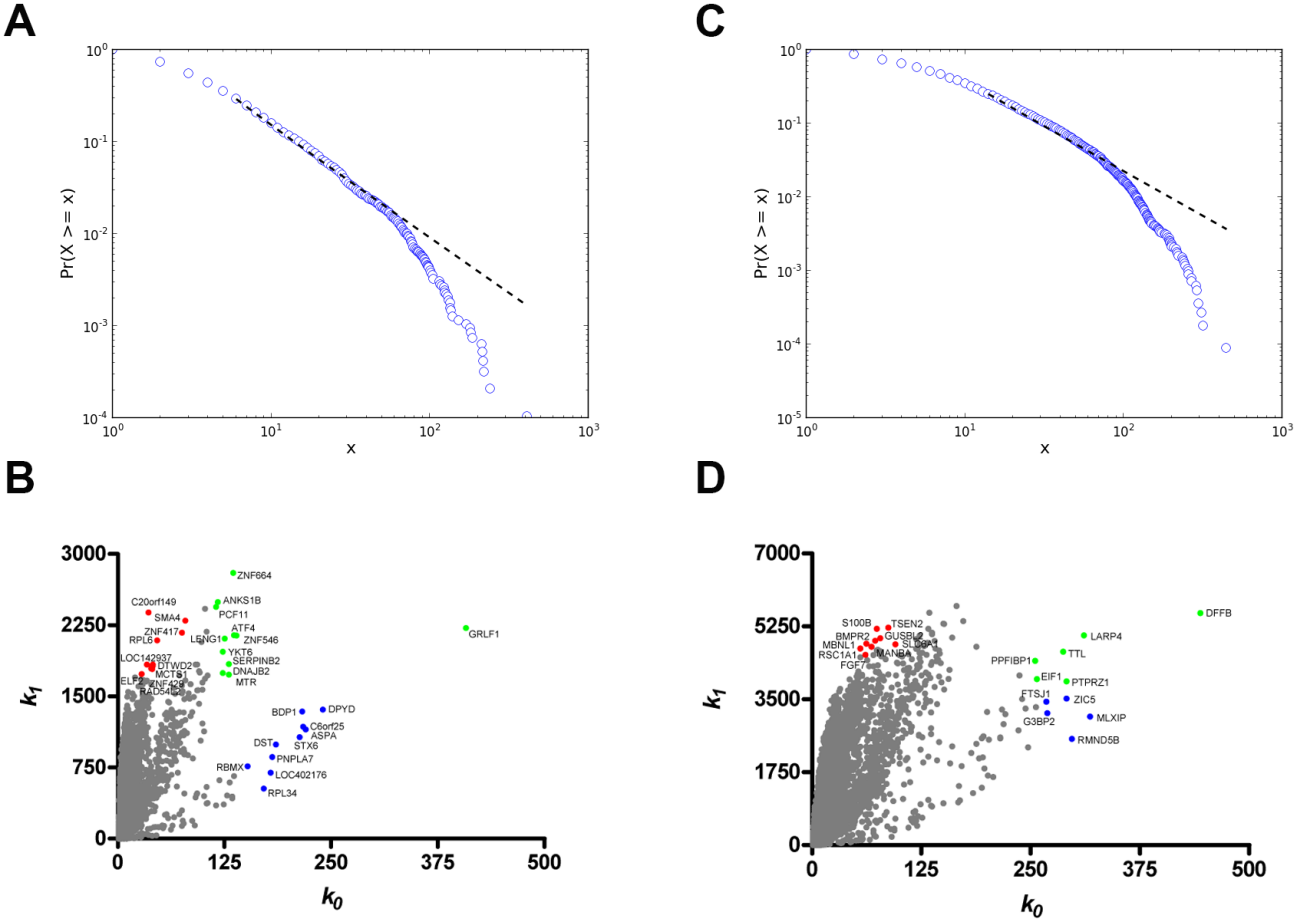
Node distribution and categorization for CO networks. Kolmogorov-Smirnov test for scale free status for E-CO (A) and L-CO (B) networks. Scatter plots of node degree (k0) vs concentric node degree (k1) measures of GO annotated genes in E-CO (C) and L-CO networks (D). Hubs (blue), VIPs (red) and high-hubs (green), identified by their gene symbols.

In the E versus L comparison, 761 DE genes were found to be upregulated and three were downregulated in the E group. Gene coexpression networks (GCNs) were inferred for E and L groups using DE or CO (encompassing all 13,427 valid transcripts) subsets of genes through Pearson’s correlation method. A 0.965 link-strength cut-off was adopted for DE networks. The resulting DE networks had 621 genes and 1367 links for the E-DE group, or 703 genes and 3,206 links for the L-DE group. We adopted a higher link-strength cut-off (0.998) to finalize the CO networks, which had 9,578 genes and 32,807 links for the E-CO group, or 11,321 genes and 76,711 links for CT group. All networks were validated as scale-free networks as seen in a normalized degree distribution log-log plot: DE and CO networks are shown in Figs 1 and 2, respectively. In these figures the nodes (genes) are depicted in different colors corresponding to their hierarchical level: blue for hubs, red for VIPs, and green for high-hubs. Gene categorization as hubs, VIPs, and high hubs, with their corresponding *k*_*0*_ and *k*_*1*_ values and biological function appear in Table 2 (DE networks) and Table 3 (CO networks).

**Table 2:** High-hubs, hubs, VIPs and communities in Early and/or Late DE networks.

	Early				Late				
Gene	Comm^1^	Cat^2^	*K*0	*K*1	Comm	Cat	*K*0	*K*1	Gene function/product [reference]
*C1orf115*	A	Hub	27	67					Integral component of membrane (Gene ID: 79762)
*SYN2*	A	VIP	6	98					Regulation of epileptic and synaptic activity on hippocampus [67]
*AW948903*	A	VIP	10	96					Expressed sequence tag (EST) AW948903
*FAM70B*	A	VIP	10	91					Official symbol: *TMEM255B*. Integral component of membrane (Gene ID: 348013)
*FHOD1*	A	High-hub	17	110					Coordinates actin filament and microtubule alignment [67]
*TUBB2A*	A	High-hub	23	95					Beta tubulin, a major component of hippocampal microtubules [71, 72]
*C19orf28*	B	Hub	24	59					Aliase: *MFSD12*. Mediates sodium butyrate (HDAC inhibitor) inhibition of Sirtuin-2 (HDAC III)-mediated hippocampal synaptic plasticity [73, 74]
*ZDHHC23*	B	VIP	7	88					Aliase: *NIDD*. Controls surface expression of calcium-activated potassium channels (BK) [76]
*KRT14*	B	High-hub	25	121					Codes for keratin 14 and modulates Notch signaling [80]
*BRUNOL5*	C	Hub	21	54					Member of the CELF-Bruno Like family of RNA-binding proteins [52]
*SCN9A*	C	VIP	9	99					Febrile seizure associated gene coding for Na_v_ 1.7 sodium channel; Allelic variants may cause FS epilepsy [46, 47]
***RTN2***	C	VIP	10	89	C	VIP	25	407	Regulates the trafficking and function of glutamate transporter EAAC1 [48]
*FLJ37078*	C	High-hub	25	128					Official symbol: *SRRM3*. Serine/arginine repetitive matrix 3 (Gene ID: 222183)
*AVIL*	C	High-hub	22	84					Codes for advillin, an actin binding protein, and regulates neurite outgrowth [54, 55]
*BAIAP2*	C	High-hub	19	82					Adaptor protein IRSp53; involved in the regulation of NMDA receptor-mediated excitatory synaptic transmission [50]
*MYH14*	D	Hub	23	67					Neurite stabilization at adhesion sites [194]
*TXNRD1*	D	VIP	10	95					Regulator of cellular redox balance (protection against oxidative stress) [195]
*AA292106*	D	High-hub	23	89					Expressed sequence tag (EST) AA292106
*C1orf92*	E	Hub	24	68					Aliase: *LRRC71*. Protein harboring a leucine-rich repeat motif (Gene ID: 149499)
*BF083139*	E	VIP	8	89					Expressed sequence tag (EST) BF083139
*KNDC1*	F	Hub	28	47					Codes for a v-KIND domain containing protein involved in the control of dendrite arborization patterns [196]
***B4GALT2***	G	Hub	28	42	D	High-hub	57	496	Major regulator of glycan synthesis involved in neuronal development and neuron outgrowth [197, 198]
*GTF3C1*	H	High-hub	21	109					Aliase: *TFIIIC*. Regulates the rearrangement of nuclear architecture allowing the coordinated expression of activity-dependent neuronal genes [82]
*EGR4*	I	Hub	27	72					Mediates BNDF induction of neuronal KCC2 transcription [56]
*B3GALNT1*	I	VIP	8	97					Glycosylation of HCN1 channels upon seizure activity in hippocampus [61]
*KCNC3*	I	VIP	7	95					Potassium channel related to febrile seizures and synaptic excitability [58, 59, 60]
***MTA1***	I	High-hub	29	137	H	VIP	27	451	Codes for a protein which is an integral part of the nucleosome remodeling and histone deacetylation complex [62]
*KIAA1539*					A	Hub	48	380	Official symbol: *FAM214B*. Family with sequence similarity 214, member B (Gene ID: 80256)
*FAM116B*					A	Hub	45	308	Aliase: *DENND6B*. DENN/MADD domain proteins regulate Rab-mediated trafficking role in neurite formation [199, 200]
*SNX25*					A	Hub	40	268	Codes for Sorting Nexin 25; modulates TGF-beta signaling pathway and is involved in epileptogenesis and TLE [106]
*VTI1B*					A	Hub	37	368	Non-canonical SNARE molecule involved in synaptic vesicle recycling and spontaneous neurotransmitter release [201]
*TMEM2*					A	Hub	34	294	Transmembrane protein 2 (Gene ID: 23670)
*ARPC5L*					A	High-hub	51	548	Arp2/3 complex protein involved in actin polymerization and control of neurite outgrowth of hippocampal neurons [107, 108]
*RLBP1*					B	Hub	43	210	Retinaldehyde binding protein 1, a molecule controlling *PAX6* (candidate genefor epilepsy) expression [110, 111]
*LRRC56*					C	VIP	24	478	Leucine rich repeat containing 5 (Gene ID: 115399)
*RACGAP1*					C	VIP	28	462	Constituent of the IQGAP1–filamin-A—RacGAP1 pathway that coordinates directional cell migration [99]
*C16orf59*					C	VIP	29	455	Chromosome 16 open reading frame 59 (Gene ID: 80178)
*TCEA2*					C	VIP	30	446	Aliase: *TFIIS*. SII class transcription elongation factor; prevents cellular death due to oxidative DNA damage [88]
*TUBB*					C	VIP	23	440	Tubulin beta I; cytoskeleton protein aberrantly expressed in the hippocampus of TLE patients [75]
*CACNA1C*					C	VIP	30	439	L-type voltage-gated calcium channel Cav1.2. Involved in synaptic activity-dependent gene expression [83]; neurotransmitter release in hippocampal interneurons [80]; control of neurite extension [85]
*LAMA5*					C	VIP	26	453	Hippocampal laminin matrix essential for its dynamic structure and for neuronal survival under stress conditions [92]
*RING1*					C	VIP	24	429	Promotes transcriptional activation/silencing via Polycomb [87]
*CALM3*					C	VIP	25	422	Calcium signal transducer involved in the NFKB activation pathway [94]
*EFHD2*					C	VIP	21	411	Conserved calcium binding protein that regulates F-actin access to cofilin [96]
*FDXR*					C	VIP	22	408	Maintenance of cytosolic and mitochondrial iron homeostasis [89]
*LRFN3*					C	VIP	23	400	Aliase: *SALM4*. Leucine-rich repeat and synaptic adhesion-like molecule; promotes neurite outgrowth and branching [202]
*ST7*					C	High-hub	56	593	Cytoplasmic protein involved in remodeling extracellular matrix structure [203]
*ANKRD39*					C	High-hub	42	586	Ankyrin repeat domain 39 (Gene ID: 51239)
*C1orf93*					C	High-hub	41	570	Official symbol: *FAM213*. Family with sequence similarity 213, member B (Gene ID: 127281)
*TESC*					C	High-hub	46	561	Calcineurin B-like protein involved in rapid hippocampal network adaptation to recurring synchronous activity [93]
*C3orf39*					C	High-hub	47	544	*POMGNT2;* aliase: *GTDC2*. Glycosyltransferase catalyzing GlcNAcylation o fO-mannosylated α-DG in the ER [204]
*CLDN10*					C	High-hub	43	521	Tight junction protein which mediates cell adhesion [205]
*ZMYM3*					C	High-hub	41	496	Transcriptional repressor and component of HDAC complexes; abundantly expressed in the brain [206]

^1^Community

^2^ Node category; Bold indicates genes present in both E and L networks, but belonging to different communities.

**Table 3:** High-hubs, hubs, VIPs and community in Early and/or Late CO networks.

	Early				Late				
Gene	Comm^1^	Cat^2^	*K*0	*K*1	Comm	Cat	*K*0	*K*1	Gene function/product [reference]
*DPYD*	B	Hub	240	1361					Dihydropyrimidine dehydrogenase involved in pyrimidine catabolism and modulation of beta-alanine production [146]
*STX6*	B	Hub	213	1072					t-SNARE family member that regulates intracellular membrane trafficking [149]
*DST*	B	Hub	185	994					Cytoskeletal-associated protein essential for maintaining neuronal cytoskeleton organization [150]
*BDP1*	C	Hub	216	1342					Aliase: *TFIIIB*. Subunit of the TFIIIB transcription initiation complex; essential component of human TFIIIC activity [195,110]
*ZNF417*	C	VIP	75	2171					Zinc finger protein (Gene ID: 147687)
*ZNF429*	C	VIP	41	1821					DNA-binding protein associated with analgesic onset in humans [207]
*MCTS1*	C	VIP	40	1788					Aliase: *MCT1*.Carboxylate transporter; its deficiency was observed in human epileptogenic hippocampus [115]
*GRLF1*	C	High-hub	408	2221					Aliase: *ARHGAP35*. Rho-GTPase involved in the promotion of neurite outgrowth [119]
*ANKS1B*	C	High-hub	117	2494					Aliase: *AIDA1*.Postsynaptic scaffolding protein linking signal events occurring at neuronal synapse with global changes in gene expression [118]
*PCF11*	C	High-hub	115	2443					3’-end processing factor enhancing RNA polymerase II nascent RNA degradation and transcriptional termination [120]
*C6orf25*	D	Hub	217	1182					Aliase: *G6bB*. Inhibitory platelet receptor bearing ITAM and ITIM motifs; modulates microglia-neuron interaction[126, 127]
*PNPLA7*	D	Hub	181	862					Patatin-like 7hospholipase involved in lipid/energy homeostasis and axonal and synaptic integrity [129, 130,127]
*C20orf149*	D	VIP	36	2384					Aliase: *PPDPF*. Pancreatic progenitor cell differentiation and proliferation factor (gene ID 79144)
*RPL6*	D	VIP	46	2092					Ribosomal protein L6 regulates HDM2—p53 pathway in response to ribosomal stress [124]
*LOC142937*	D	VIP	34	1837					Uncharacterized gene products BC008131 (Gene ID: 142937)
*DTWD2*	D	VIP	41	1837					Prothymosin alpha involved in response to oxidative stress and neuronal survival [125]
*ZNF664*	D	High-hub	135	2800					Zinc finger protein 664 (Gene ID: 144348)
*ZNF546*	D	High-hub	136	2144					Zinc finger protein 546 (Gene ID: 339327)
*DNAJB2*	D	High-hub	123	1747					Aliase: *HSP70*. heat-shock protein expressed in hippocampal neurons; stress marker of TLE [121]
*MTR*	D	High-hub	130	1728					Regulation of homocysteine, an excitatory amino acid, protecting hippocampal neurons against oxidative stress [122, 123]
*LOC402176*	E	Hub	179	697					Aliase: *RPL21*. Ribosomal protein L21 (Gene ID: 6144)
*ASPA*	H	Hub	220	1150					Aspartoacylase, catabolizes NAA (N-acetyil-L-aspartic acid) in oligodendrocytes; responsive to glutamatergic activity [144]
*SMA4*	H	VIP	79	2299					Aliase: *SMN1*. SMN protein that modulates neuronal survival [132]
*RAD54L2*	H	VIP	39	1793					Aliase: *ARIP4*. Chromatin remodeling factor; modulates excitation/inhibition balance in hippocampal neurons towards seizure inhibition [133]
*ELF2*	H	VIP	28	1737					E1f transcription factor;promotes cell survival under cytokine stress [134]
*ATF4*	H	High-hub	139	2142					Transcription factor mediating neuronal resistance against oxidative stress [136, 137]
*LENG1*	H	High-hub	125	2109					Leukocyte receptor cluster (LRC) member 1 [140]
*YKT6*	H	High-hub	123	1970					Conserved SNARE essential for ER-Gogi transport, highly expressed in neurons and enriched in hippocampus [141]
*SERPINB2*	H	High-hub	130	1840					Synaptic activity-induced neuroprotection against seizure-induced brain-damage [143]
*RPL34*	J	Hub	171	529					Ribosomal protein L34, a Cdk5 inhibitor [151]
*RBMX*	S	Hub	152	764					RNA binding motif protein involved in the promotion of neurite growth [154]
*MLXIP*					B	Hub	318	3089	Aliase: *MondoA*. glucose sensing transcription factor involved in glucose homeostasis [178]
*ZIC5*					C	Hub	291	3523	C2H2-type zinc finger protein involved in neuronal development and inhibition of neuronal differentiation via Notch [208]
*S100B*					C	VIP	74	5192	Astrocyte-derived cytokine; promotes neurite outgrowth; increases intracellular calcium in hippocampal neurons [155]
*GUSBL2*					C	VIP	78	4968	Aliase: *GUSBP4*. Glucuronidase beta pseudogene 4 (Gene ID: 375513)
*BMPR2*					C	VIP	72	4907	Bone morphogenetic protein receptor 2; involved in astrocyte development and survival/differentiation of GABAergic and dopaminergic neurons [165]
*MBNL1*					C	VIP	62	4837	Regulatory splicing factor involved in the splicing of the microtubule-associated protein Tau [169]
*MANBA*					C	VIP	68	4764	Glycosyl hydrolase 2 family (Gene ID: 4126)
*RSC1A1*					C	VIP	55	4723	Regulates neuronal expression of the Na+-D-glucose cotransporter SGLT1; increased in the hippocampus during epileptic seizures [172]
*FGF7*					C	VIP	61	4568	Fibroblast growth factor 7; essential for inhibitory synapse formation in the hippocampus [173]
*DFFB*					C	High-hub	444	5575	Aliase: *DFF40*. Triggering of DNA fragmentation, an early event in apoptotic neuronal cell death after brain injury [175, 176]
*LARP4*					C	High-hub	311	5039	La-related protein 4; interacts with poly(A)-binding protein and promotes mRNA homeostasis [177]
*EIF1*					C	High-hub	257	3987	Eukaryotic translation initiation factor 1 [178]
*PTPRZ1*					C	High-hub	291	3935	Receptor-type protein tyrosine phosphatase; it's concentration is increased in the hippocampus of MTLE patients [174]
*G3BP2*					D	Hub	269	3168	Ras-GTPase activating protein; contributes to stress granule formation [180]; expression levels altered in TLE [181]
*FTSJ1*					D	Hub	268	3447	RNA methyltransferase expressed in the hippocampus; associated to seizures [182]
*RMND5B*					F	Hub	297	2551	Aliase: *GID2*. E3 ubiquitin ligase involved in the catabolic-induced degradation of gluconeogenic enzymes [157]
*TSEN2*					F	VIP	87	5225	tRNA splicing endonuclease complex subunit; associated with seizures [159]
*SLC6A1*					F	VIP	95	4821	Aliase: *GAT1*. GABA transporter 1; a major GABA transporter in the brain [156]; hippocampal expression increased in TLE patients [153]
*TTL*					G	High-hub	287	4647	Tubulin-tyrosine-ligase; involved in neuronal organization and control of neurite extensions [184]
*PPFIBP1*					G	High-hub	255	4425	Liprin-family scaffold protein regulating cell adhesion, cell migration and synapse development [185]

^1^Community

^2^ Node Category

#### Connectivity

E networks exhibited lower connectivity when compared to L networks. The k values for these four networks were: E-DE = 4.40; E-CO = 6.85; L-DE = 9.12; L-CO = 13.55.

#### Community detection

**Fig 3:**
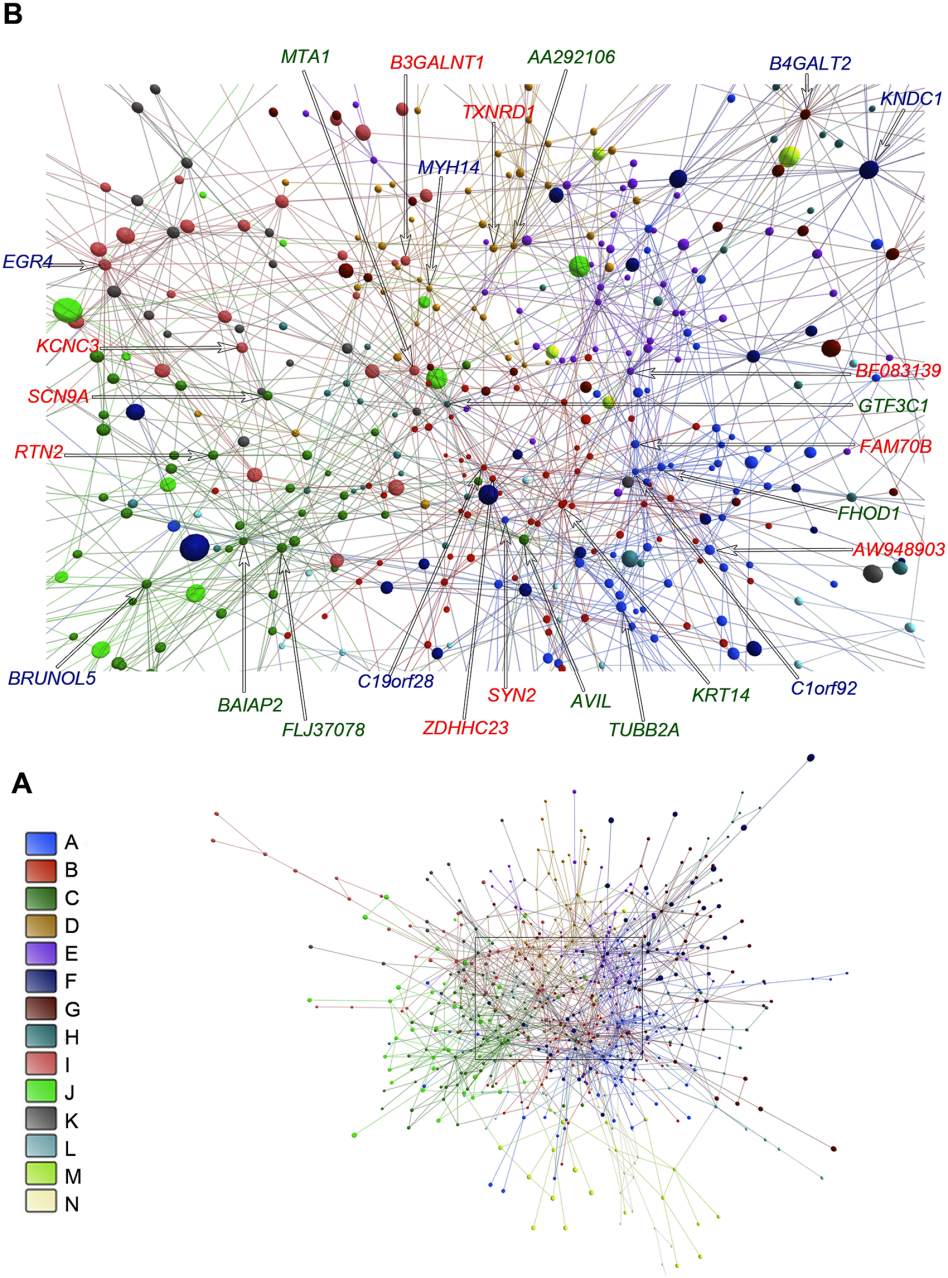
Community analysis for E-DE network. The communities are indicated by different colors (A). Highly connected nodes (B) occupy central positions and their correspondent GO gene symbols are depicted in different colors corresponding to their hierarchical level: blue for hubs, red for VIPs, and green for high-hubs. In amplification B node size is not related to node degree.

**Fig 4:**
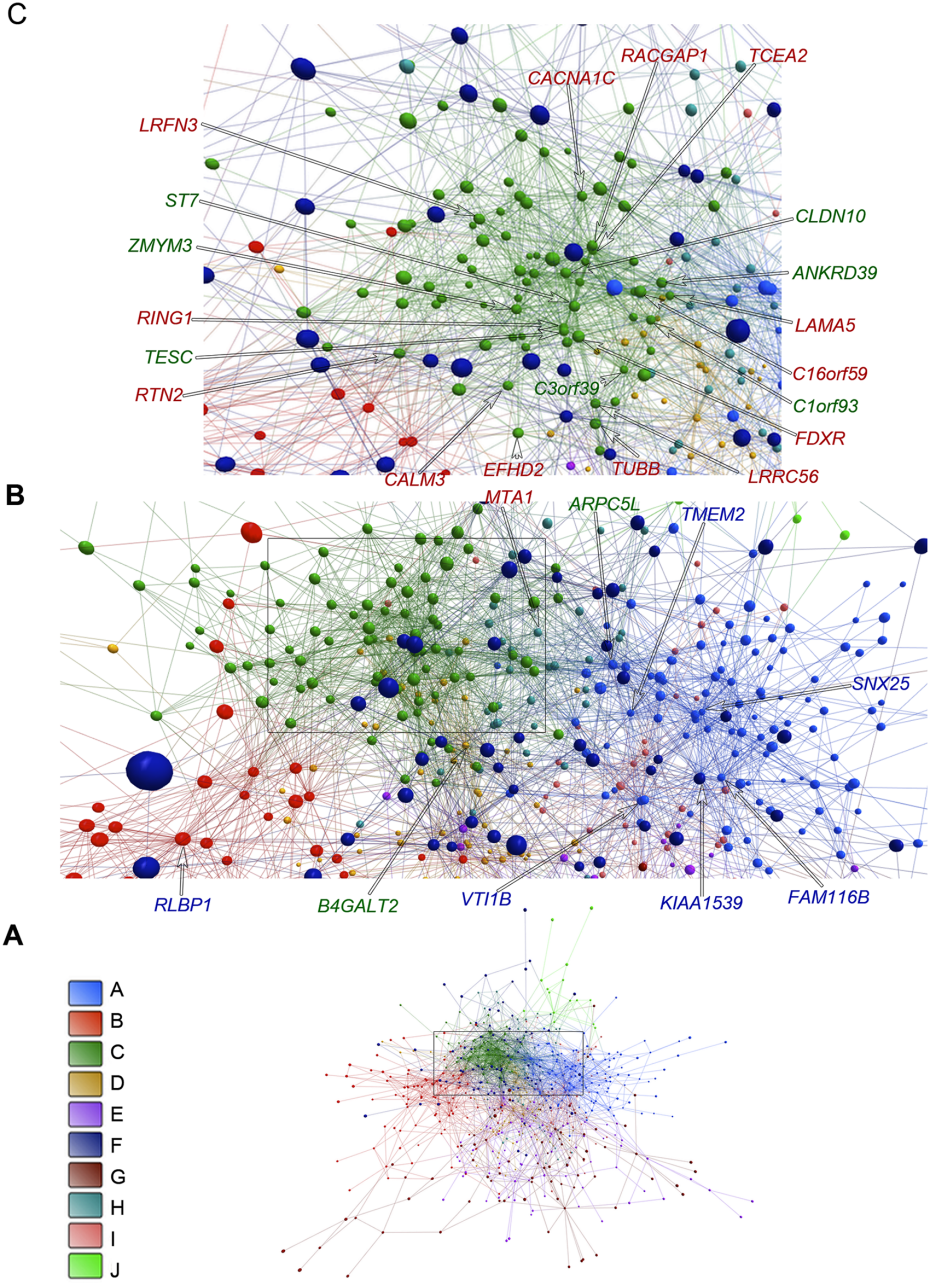
Community analysis for L-DE network. The communities are indicated by different colors (A). Highly connected nodes (B-C) occupy central positions and their correspondent GO gene symbols are depicted in different colors corresponding to their hierarchical level: blue for hubs, red for VIPs, and green for high-hubs. In the amplifications B-C node size is not related to node degree.

**Fig 5:**
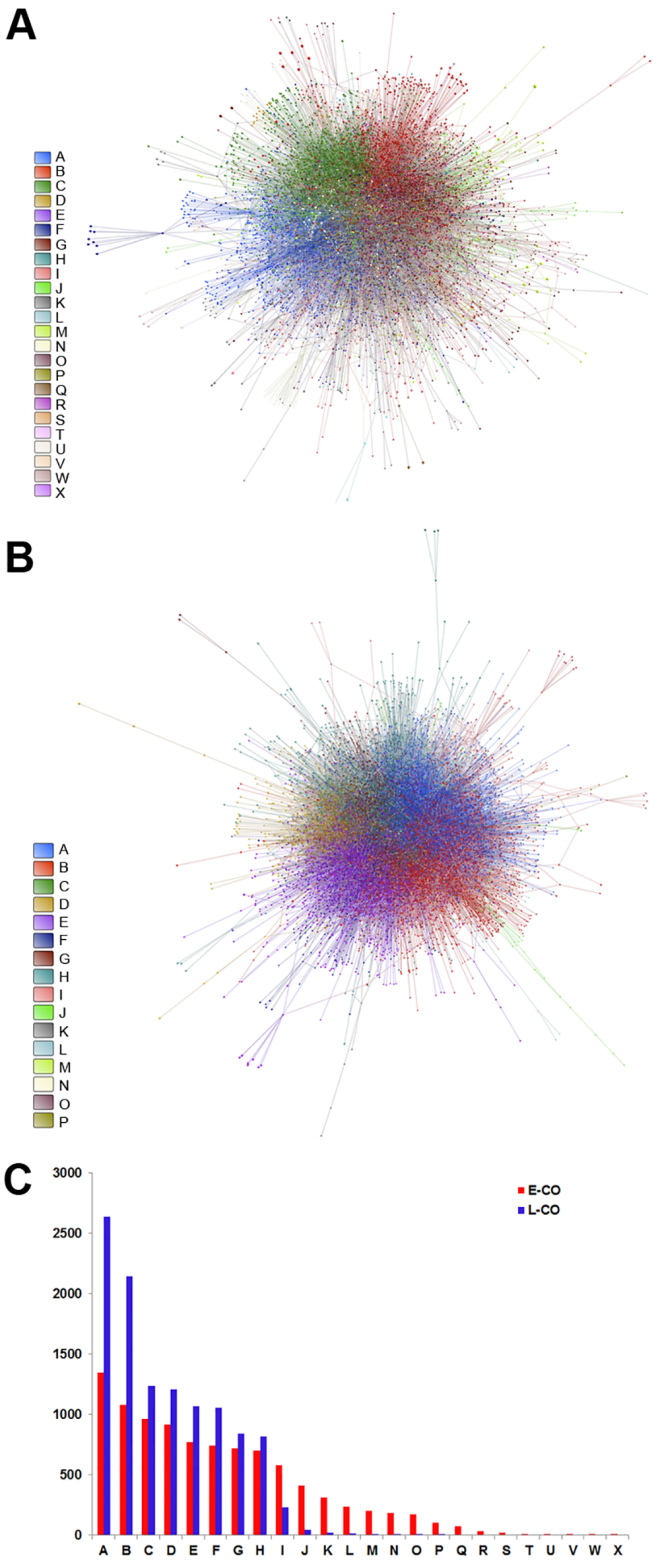
Community analysis for CO networks. The communities are indicated by different colors for E-CO (A) and for L-CO (B). Fig **5**C shows the number of nodes per community in each of the CO networks (indicated by red and blue bars for E-CO and L-CO, respectively).

An overall picture of DE gene communities (modules) is depicted in Fig 3A for E-DE and in Fig 4A for L-DE networks. Different node colors identify the distinct gene communities in each network. Fig 3B, Fig 4B and 4C present, respectively, E-DE and L-DE hierarchy-categorized selected nodes identified by their corresponding GO gene symbols. Symbol letter colors indicate hubs (blue), VIPs (red) or high hubs (green). E-CO and L-CO networks, respective communities, and hierarchy-categorized selected nodes can be properly visualized only in 3D and are shown in S1 and S2 Videos. DE and CO networks presented good quality of community structure and modularity values were quite similar for all networks: E-DE = 0.657; L-DE = 0.530; E-CO = 0.514; L-CO = 0.506. It is interesting to note that the E-CO network has more gene communities (24) than the L-CO network (16) as depicted in Fig 5A and 5B respectively. Fig 5C shows the number of nodes per community in each of the CO networks. E networks (DE and CO) have lower connectivity and their communities are more sparsely connected, what may indicate a higher grade of dysregulation in cell’s functional organization [4, 43]. A set of simulations run with slightly different link-strength thresholds (from 0.930 up to 0.980 for DE networks, and from 0.990 up to 0.998 for CO networks) did not reveal alterations in community structure, thus indicating its robustness.

### Community structure analysis of transcriptional networks

**Fig 6:**
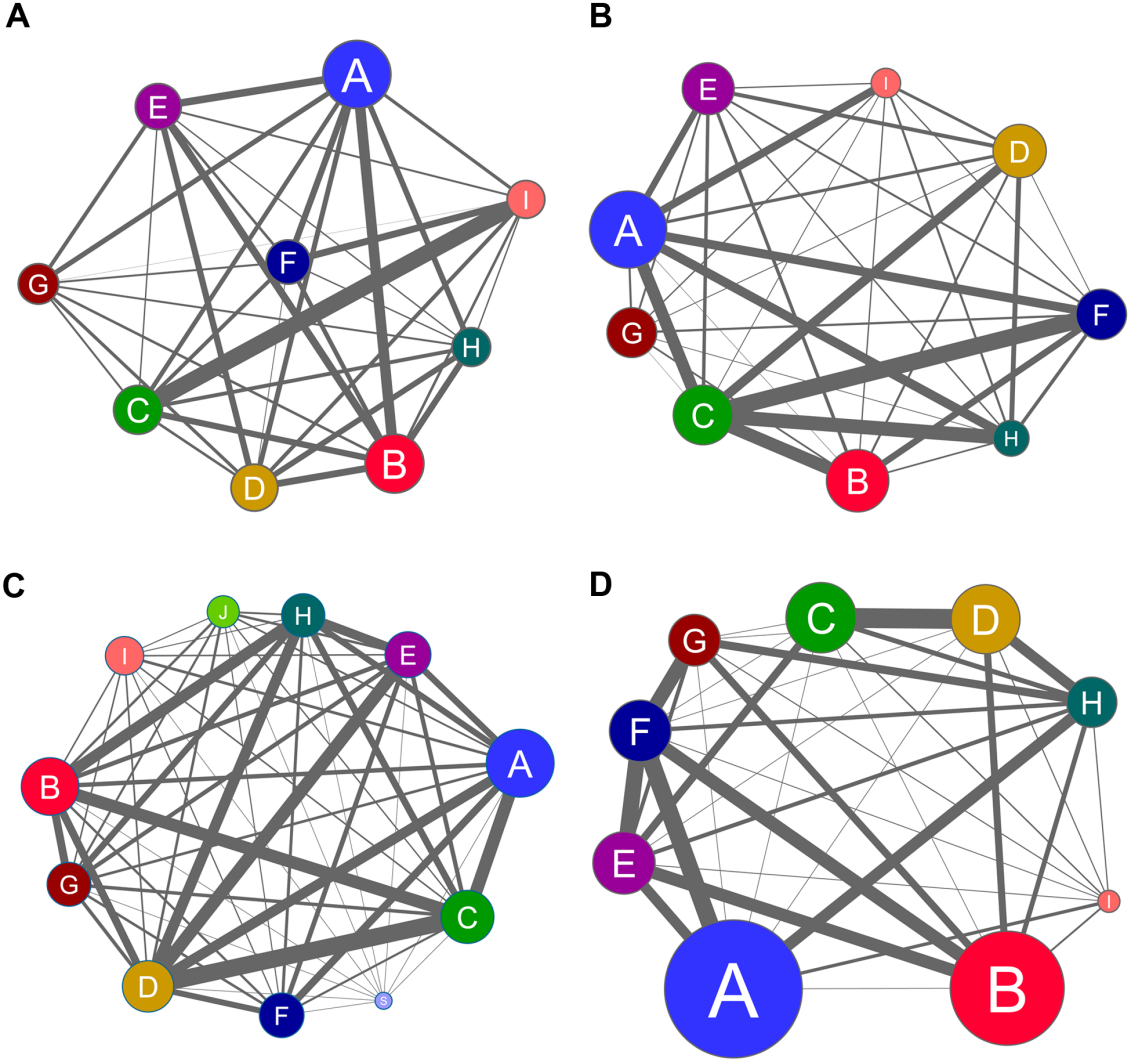
CGCS summarizing the relationships among the communities. CGCS is depicted for E-DE (A), L-DE (B), E-CO (C) and L-CO (D) networks. The edge width is proportional to the fraction of edges linking distinct communities. The node size is proportional to the number of nodes in each community. In each of the four networks only the top nine communities in number of nodes (A to I) were considered for this analysis, except for communities J and S in the E- CO network (C), since these communities contain relevant hubs.

This section portrays the biological functions of selected hubs, VIPs and high- hubs in the context of the distinct gene communities, i.e. transcriptional modules, found in DE and CO networks generated for E and L groups. Coarse-grained community structure (CGCS) was obtained for each network, yielding the relationships between each community in the network (Fig 6). Communities with the strongest connection weights (fraction of edges linking distinct communities) hold the most significant functional interactions in the network [30, 44, 45]. Therefore, the subsequent analysis of gene communities in DE and CO networks was performed considering not only the gene/node hierarchy but, and principally, the networks’ CGCS.

#### E-DE network

This network encompasses 14 gene communities, of which 10 contain high hierarchy nodes/genes—categorized as hubs, VIPs or high-hubs—with high network centrality (Fig 3A, 3B and Table 2). Most of these high hierarchy genes play relevant roles in epilepsy and brain functioning. These roles will be analyzed below in the setting of gene community relationships. In the E-DE network communities C and I have the strongest connection weights (Fig 6A), followed by communities A and B.

Community C harbors six E-DE high hierarchy genes and four of them are related to neuronal excitability. Two of these genes are VIPs: *SCN9A*, which codes for Na_v_1.7 sodium channel and whose allelic variants are implicated in genetic epilepsies with febrile seizures [46, 47] and *RTN2*, also a VIP in L-DE network module C, a regulator of the trafficking and function of glutamate transporter EAAC1 (excitatory amino acid carrier 1) [48]; dysregulation of EAAC1 was reported in experimental models of epilepsy and also in the hippocampus of temporal lobe epilepsy (TLE) patients [49]. The third gene, *BAIAP2*, is a high-hub. This gene codes for an adaptor protein (IRSp53) involved in the regulation of NMDA receptor-mediated excitatory synaptic transmission, long-term potentiation, and learning and memory [50]. Rapid surface accumulation of NMDA receptors in dentate gyrus and CA3 pyramidal cells increases glutamatergic excitation during status epilepticus [51]. The fourth gene in this functional group, *BRUNOL5*, aliase *CELF5*, codes for a member of the CELF-Bruno Like family of RNA-binding proteins. *BRUNOL5* expression is restricted to brain [52]. CELF proteins share structural and functional redundancy. Human BRUNOL4, closely related to BRUNOL5 [48], is involved in fine-tuning synaptic transmission and its deficiency causes recurrent seizures in mice and man [53].

The remaining two genes in community C are high-hubs: *AVIL* codes for advillin, an actin binding protein, and regulates neurite outgrowth [54, 55]; and *FLJ37078* encodes a hitherto uncharacterized protein.

In community I all four high hierarchy genes are clearly related to febrile seizures, synaptic activity and epilepsy. *EGR4*, a hub, mediates BNDF induction of neuronal KCC2 (potassium chloride cotransporter 2) transcription [56]. *KCC2* variants determine susceptibility to febrile seizures [57]. *KCN3*, a VIP, codes for a potassium channel related to febrile seizures and synaptic excitability [58, 59, 60]. *B3GALTN1*, *also a VIP*, glycosylates and promotes heteromerization of HCN1-HCN2 channels in hippocampus upon seizure activity, thus enhancing network excitability [61]. *MTA1*, a high-hub, and a VIP in L-DE community H, codes for a core component of NuRD (Nucleosome Remodeling and histone Deacetylation) complex [62], where it enhances histone deacetylases (HDACs) 1 and 2 activity in order to repress gene expression [63]. *HDAC2*—the target of the antiepileptic drug Valproate—is overexpressed in the brain of TLE patients and its transcriptional repression activity plays an important role in the pathogenesis of epilepsy [64].

Communities A and B have the second strongest connection weights in E-DE. Noteworthy, community A harbors *SYN2*, a VIP that encodes Synapsin II, a phosphoprotein which desynchronizes neurotransmitter release at inhibitory synapses by interacting with presynaptic Ca channels, modulating synaptic transmission and plasticity; allelic variants of *SYN2* contribute to epilepsy predisposition [65, 66]. In community A are also located two high-hubs—*FOHD1* and *TUBB2A*—related to microtubule function and structure. *FOHD1* coordinates actin filament and microtubule alignment to mediate cell elongation [67], being possibly involved in hippocampal neuronal polarization [68, 69]. There is a loss of normal neuronal polarization in temporal lobe epilepsy [70]. *TUBB2A* codes for beta tubulin, which is a major component of hippocampal microtubules [71, 72].

Community B includes three genes acting in several epilepsy pathways. *C19orf28* (aliase *MFSD12*) is a hub and its gene product (protein pp3501) mediates sodium butyrate (HDAC inhibitor) inhibition of Sirtuin-2 (HDAC III)-mediated hippocampal synaptic plasticity [73, 74]. Sirtuin-2 expression is decreased in mesial temporal lobe epilepsy (MTLE) [75]. *ZDHHC23* (aliase *NIDD*) is a VIP and codes for a DHHC-containing protein involved in: i) regulation of cell surface expression of calcium-activated potassium (BK) channels [76]; ii) regulation of NO signaling pathways at postsynaptic sites [77]; iii) control of neuronal hiperexcitability via BK channels [78]. Interestingly, a seizure—related down regulation of BK channel protein levels was described in the pilocarpine model of temporal lobe epilepsy [79], indicating a role for BK and NIDD in MTLE. The high hub *KRT14*, codes for keratin 14 and modulates Notch signaling [80]. Notch signaling is up-regulated in temporal lobe epilepsy (reviewed in [81]).

The remaining communities and respective high hierarchy genes are described in Table 2. Here is worth to mention here community H, which harbors *GTF3C1* (aliase *TFIIIC*) a gene that regulates the rearrangement of nuclear architecture allowing the coordinated expression of activity-dependent neuronal genes [82]. This gene controls the expression of *BAIAP2*, which is high hub in E-DE community C.

In synthesis, it is possible to say that communities C and I, which share the strongest connection weights, include genes closely related to neural excitability and febrile seizures mechanisms, with relevance for *SCN9A* and *KCCN3*, implicated in febrile seizures and synaptic excitability, followed by the regulators of synaptic excitability (RNT2, BAIAP2 and B3GALTN1) and by *MTA1*, the enhancer of HDAC2 and a Valproate target. Several of the high hierarchy genes in A and B communities are also related to neuronal excitability, or to genomic mechanisms favoring this mechanism. The implications of these results for the development and onset age of MTLE in patients with febrile seizure history will be discussed later in this paper.

#### L-DE network

This network (Fig 4A–4C) has 10 gene communities, of which 5 (Table 3) contain high hierarchy nodes (hubs, VIPs or high-hubs). Here the strongest connection weights are centered in module C, which also contains most of the VIPs and high-hubs of the L-DE network, which are involved in relevant epilepsy mechanisms, as follows.

Two community C VIPs, *RTN2* and *CACN1C*, are significantly linked to neuronal excitability. *RTN2*, also a VIP in E-DE network module C, is a regulator of the glutamate transporter EAAC1 [48]; the dysregulation of EAAC1 was reported in animal models of epilepsy and in the hippocampus of TLE patients [49]. *CACNA1C* encodes the alpha 1C subunit of the L-type voltage-gated calcium channel Cav1.2 which plays relevant roles in: i) synaptic activity-dependent gene expression [83] ii) regulation of neurotransmitter release in hippocampal interneurons [84]; iii) control of neurite extension [85]. Here is very important to note that Cav1.2 calcium channels are temperature- sensitive and support the intrinsic firing of pyramidal neurons during hyperthermia, thus providing a target for the treatment of febrile seizures [86].

The VIPs *RING1*, *TCEA2*, *FDXR*, *LAMA5*, and the high-hub *TESC*, are associated to mechanisms of stress response and brain homeostasis. *RING1* promotes transcriptional activation/silencing via Polycomb [87]. *TCEA2*, codes for a SII class transcription elongation factor that plays an important role in preventing cell death due to oxidative DNA damage [88]. *FDXR* codes for the sole human ferredoxin reductase; it is involved in the maintenance of cytosolic and mitochondrial iron homeostasis [89], and in cell sensitization to oxidative stress and apoptosis in TLE [90, 91]. *LAMA5* codes for a component of hippocampal laminin matrix essential for its dynamic structure and for neuronal survival under stress conditions, such as excitotoxicity [92]. *TESC* codes for a Ca2+ binding calcineurin B-like protein involved in rapid hippocampal network adaptation to recurring synchronous activity [93].

The other high hierarchy genes community C with functions associated to epilepsy-related mechanisms are: i) the VIP *CALM3*, that encodes a calcium signal transducer involved in the NFKB activation pathway [94], which is dysregulated in hippocampal tissues of TLE patients [95]; ii) the high-hub *EFHD2*; its gene product is a conserved calcium binding protein that regulates F-actin access to cofilin [96], influencing actin cytoskeleton remodeling and the excitability of epileptic hippocampus [97], as well as astrogial loss following status epilepticus [98]; iii) the VIP *RACGAP1* a constituent of the IQGAP1—filamin-A—RacGAP1 pathway that coordinates directional cell migration [99], which is frequently aberrant in epileptic hippocampus [70, 100].

Although strongly connected with community C, community F (Fig 6B) does not harbor high hierarchy genes within the *k*_*0*_ and *k*_*1*_ cut-off values adopted for L-DE network (see Fig 1D). A scatter plot of node degrees for module F genes and the high hierarchy genes of L-DE appear in S2 Fig Among the module F highly connected genes are: i) *CNTNAP1*, which encodes contactin associated protein1, a member of the contactin-PTPRZ1 complex that regulates the traffic and synaptic content of AMPA glutamate receptor subunit GluA1 in hippocampal neurons [101]; ii) *CCNE1*, a Sirtuin-2 regulator [102] and iii) *PKIG* which encodes a protein kinase involved in endothelial barrier function [103]. Remarkably, GluA1 hippocampal expression is altered during status epilepticus [101, 104], MTLE patients show reduced hippocampal expression of Sirtuin-2 [75], and endothelial barrier function is altered in epilepsy [105].

The other significant connections of community C (Fig 6B) occur with community A—where are located *SNX25* and *ARPC5L*, markers of intractable epilepsy—and with communities H and B which harbor, respectively, the genes *MTA1* (a high-hub in E-DE community I, functionally described above) and *RBPL1*, regulators of two epilepsy-associated genes (*HDAC2* and *PAX6*, targets of the antiepileptic drug Valproate). The hub *SNX25* codes for Sorting Nexin 25, a PX domain protein which modulates TGF-beta signaling pathway and is involved in epileptogenesis and TLE development [106]. *SNX25* is a biomarker of intractable epilepsy, being overexpressed in TLE patients [106]. *ARPC5L* encodes an Arp2/3 complex protein involved in actin polymerization [107] and in the control of neurite outgrowth of hippocampal neurons [108]. Arp2/3 expression is increased in the temporal lobe cortex of intractable epilepsy patients [109]. *RLBP1* encodes the retinaldehyde binding protein 1, a retinoic acid (RA) signalling molecule. It is noteworthy that RA-signalling promotes the expression of *PAX6* [110], which is a candidate gene for epilepsy [111] and whose expression on neuronal cells may be altered by Valproate [112].

The overall picture that emerged from the analysis of community relationships in L-DE is somewhat different from that obtained for E-DE. There is a quite dissimilar functional scenario and also different key players (excepting for *RTN2*, *B4GALT2* and *MTA1*). Here community C—which centers the strongest connection weights,—harbors most of the VIPs and high-hubs, what indicates that this community is essential for keeping L-DE network stability and functionality. Contrarily to the C-I excitability axis in E-DE, only part of the genes in L-DE community C, such as *CACNA1C* and *RNT2*, is associated to neuronal excitability. In fact, a sizable number of genes are related to stress response and brain homeostasis, and others to different epilepsy-associated mechanisms, including those related to cytoskeleton and cell migration. The communities F and A, both strongly connected with community A, harbor genes involved in different biological process with relevancy for epilepsy, like *CNTAP1*, a regulator of GluA1, and the two markers of intractable epilepsy, *SNX25* and *ARPCL5*. The community detection data for L-DE and E-DE networks will be considered comparatively in the Discussion section.

#### E-CO network

The complete gene coexpression network for the E group encompasses 24 gene communities of which only 7 contain high hierarchy nodes (Figs 2B and 5A) with high network centrality (S1 Video). In this network the strongest connection weights involve communities D and C, followed by communities H and B (Fig 6C). It is noteworthy that communities C, D and H concentrate all the VIPs and high-hubs of E-CO network. The summarized functional description of all high hierarchy genes contained in E-CO appears in Table 3.

Community C includes five high hierarchy genes related to brain homeostasis and regulation of neuronal gene expression: *BDP1*, *MCTS1*, *ANKS1B*, *GRLF1* and *PCF11*. *BDP1* (aliase *TFIIIB*) is a hub and codes for a subunit of the TFIIIB transcription initiation complex, being an essential component of human TFIIIC activity [113, 114]. TFIIIC regulates the coordinated expression of activity-dependent neuronal genes, such as *BAIAP2* [82], a high-hub in E-DE community C. The VIP *MCTS1* (aliase *MCT1*) codes for a carboxylate transporter and its deficiency was observed in human epileptogenic hippocampus [115]. *MCTS1* is involved in TLE (and especially in MTLE) by influencing brain energy homeostasis, mitochondrial function GABAergic and glutamatergic transmission and flux of lactate through the brain [116, 117]. *ANKS1B* (aliase *AIDA1*), a high-hub, codes for a postsynaptic scaffolding protein that links persistent signal events occurring at neuronal synapse with global changes in gene expression [118]. The high-hubs *GRLF1* (aliase *ARHGAP35*) and *PCF11* codes, respectively, for a Rho-GTPase involved in the promotion of neurite outgrowth [119] and for a 3’-end processing factor that enhances RNA polymerase II nascent RNA degradation and transcriptional termination [120].

The high hierarchy genes of community D are mostly involved in responses to stress and neuronal survival. Two high-hubs, *DNJB2* and *MTR* have relevant roles in this task. *DNJB2* (aliase *HSP70*), codes for a heat-shock protein expressed in hippocampal neurons. HSP70 is a well-known stress marker of TLE; its expression before insult improves neuronal survival [121]. *MTR* codes for the enzyme 5-methyltetrahydrofolate-homocysteine methyltransferase. This enzyme regulates the brain levels of homocysteine, an excitatory amino acid, protecting hippocampal neurons against oxidative stress [122, 123]. Two VIPs are also involved in responses to stress: *RPL6* whose gene product is ribosomal protein L6, a regulator of the HDM2 —p53 pathway in response to ribosomal stress [124]; and *DTWD2*, coding for prothymosin alpha, a highly acidic nuclear protein of the alpha-thymosin family involved in response to oxidative stress and neuronal survival [125]. Finally, two hubs, *C6orf25* and *PNPLA7*, are also related to protective roles. *C6orf 25* (aliase *G6bB*) encodes an inhibitory platelet receptor bearing ITAM and ITIM motifs and it is involved in the modulation of microglia-neuron interaction [126, 127, 128]. *PNPLA7* codes for a patatin-like phospholipase, structurally similar to PNPLA6, involved in lipid and energy homeostasis [129] and possibly involved in axonal and synaptic integrity [130, 131].

Community H encompasses set of three VIPs, four high-hubs and one hub, all exerting important roles in neuroprotection, modulation of neuronal excitability, and seizure inhibition. These genes are in the following described according to their hierarchic category and biological functions

VIPs: *SMA4* (aliase *SMN1*) codes for the survival motor neuron (SMN) protein and modulates neuronal survival cooperating with PP4R2 (a regulatory subunit of phosphatase 4) [132]. *RAD54L2* (aliase *ARIP4*) encodes a chromatin remodeling factor that interacts with serine/threonine kinase DIRK1A (minibrain kinase) modulating excitation/inhibition balance in hippocampal neurons towards seizure inhibition [133]. *ELF2* codes for an E1f transcription factor and promotes cell survival under cytokine stress (a condition present in MTLE) by increasing valosin-containing protein (VCP) expression [134]. Here is interesting to note that hippocampal valosin is vulnerable to oxidative stress in excitotoxin-induced neuronal injury [135].

High-hubs: *ATF4* codes for a transcription factor mediating neuronal resistance against oxidative stress [136, 137]. Interestingly, ATF4 expression is regulated by *ELF2* [138], a VIP in this community (see above) Moreover, ATF4 is involved in the differential control of hippocampal GABABR1a and GABABR1b Subunit Gene Expression through Alternative Promoters [139]. *LENG1* codes for the leukocyte receptor cluster (LRC) member 1 with uncharacterized function in brain [140]. *YKT6* codes for an evolutionary conserved SNARE essential for ER-Gogi transport, highly expressed in neurons and enriched in hippocampus [141]. *YKT6* is co-upregulated with *S100B* (a VIP in L-CO network, module C) upon seizures [142]. *SERPINB2* is one of the nine hippocampal core genes for synaptic activity-induced neuroprotection against seizure-induced brain-damage [143]. These genes are collectively termed Activity-regulated Inhibitor of Death (AID) genes. All AID genes are activated by calcium signaling [143].

Hub: *ASPA* codes for aspartoacylase, an enzyme that catabolizes NAA (N-acetyil-L-aspartic acid) in oligodendrocytes and is responsive to glutamatergic activity [144]. Decreased NAA levels in hippocampal CA3 are characteristic of MTLE [145].

Community B contains three hubs and their biological functions are detailed here. *DPYD* codes for dihydropyrimidine dehydrogenase, an enzyme involved in pyrimidine catabolism which also modulates the production of beta-alanine, a neuromodulator of inhibitory transmission in the brain [146]. Altered function of DYPD is associated with seizures [147] and intellectual disability [148]. *STX6* codes for syntaxin 6, a t-SNARE family member that regulates intracellular membrane trafficking [149]; *DST*, encodes a large multidomain cytoskeletal-associated protein essential for maintaining neuronal cytoskeleton organization [150].

Finally, also depicted in Fig 6C are communities J and S, where two relevant hubs are located. Community J harbors the hub *RPL34*, which encodes ribosomal protein L34, a Cdk5 inhibitor [151]. Cdk5 is a mediator of neuronal death and survival [152] and is involved in cell degeneration in hippocampal neurons after an excitotoxic injury [153]. Community S has the hub *RBMX*, a gene coding for a RNA binding motif protein involved in the promotion of neurite growth [154]. Neurite growth is a hallmark of TLE [155].

Altogether, E-CO network most connected communities encompass genes predominantly related to compensatory pathways in epilepsy (seizure inhibition, neuronal survival and responses to stress conditions). These genes are concentrated in communities C, D and H, which also contain all VIPs and high-hubs in this network, pointing out for the role of those genes in supporting network stability. This issue will be further developed in the Discussion section.

#### L-CO network

The complete gene coexpression network for the L group has 16 gene communities) of which only 5 contain high hierarchy nodes (Fig 2D) with high network centrality (S2 Video). In this network the strongest weight connections are centered in community F and between the communities C and D (Fig 6D). Community F contains two VIPs and one hub whose altered expression is associated with pro-epileptic effects. The most relevant of these gene is *SLC6A1* (aliase *GAT1*), which codes for GABA transporter 1 (GAT1), a major GABA transporter in the brain [156]. In the epileptogenic sclerotic hippocampus of MTLE patients the expression of GAT1 is decreased in CA3 but it is increased along granule cell dendrites [157]; inhibitors of GAT1 have been studied and developed for epilepsy control [158]. The other VIP is this community is *TSEN2*, whose gene product is a tRNA splicing endonuclease complex subunit whose mutations are associated with seizures and pontocerebellar hypoplasia [159]. The hub is *RMND5B* (aliase *GID2*), a gene that encodes an E3 ubiquitin ligase involved in the catabolic-induced degradation of gluconeogenic enzymes [160]. Gluconeogenesis occurs in astrocytes and is pro-epileptic [161].

Community C concentrates most of the VIPs and high-hubs in L-CO network. In this community all the VIPs with known biological functions are associated to seizure activity/severity, or with vulnerability to epilepsy. *S100B* is a well-known MTLE marker, which codes for an astrocyte-derived cytokine that promotes neurite outgrowth and increases the levels of intracellular calcium in hippocampal neurons [155, 162]. *S100B* coded protein is a marker of astroglial activation: its hippocampal levels were found to be higher at the side of seizure onset in patients with refractory MTLE [163] and its plasma concentration was reported to be elevated in MTLE patients [164]. *BMPR2* encodes the bone morphogenetic protein (BMP) receptor 2, involved in astrocyte development and survival and differentiation of GABAergic and dopaminergic neurons [165]. *BMPR2* was found to be strongly expressed in the hippocampal formation of human and rat adult brain [166, 167] and it is upregulated in rat adult hippocampus during neuroplasticity or repair upon brain injury [168]. *MBNL1* codes for a regulatory splicing factor involved in the splicing of the microtubule-associated protein tau [169]. Elevated brain tau levels are associated with seizure severity [170]. *MAMBA* encodes a member of the glycosyl hydrolase 2 family. The encoded protein localizes to the lysosome where it is the final exoglycosidase in the pathway for N-linked glycoprotein oligosaccharide catabolism. Mutations in this gene cause beta-mannosidosis, including severe forms with neonatal onset epilepsy [171]. *RSC1A1* gene product regulates the neuronal expression of the Na^+^-D-glucose cotransporter SGLT1, which is increased in the hippocampus during epileptic seizures [172]. *FGF7* codes for the fibroblast growth factor 7 (FGF7), which is essential for inhibitory synapse formation in the hippocampus. Mice with deficiency in this gene display mossy fiber sprouting and increased neurogenesis, becoming vulnerable to epilepsy [173].

The high-hubs in community C are either involved in mechanisms related to epilepsy pathogenesis (*DFF40* and *PTPRZ1*) or in the control of gene expression and translation initiation (*LARP4*, *EIF1*). *PTPRZ1* encodes a member of the receptor protein tyrosine phosphatase family whose concentration is increased in the hippocampus of MTLE patients [174]. This receptor is involved damaged-induced gliosis and neuronal reorganization in the hippocampus of MTLE patients [174]. *DFFB* (aliase *DFF40*) codes for a caspase-activated deoxyribonuclease (CAD)/DNA fragmentation factor 40 (DFF40) involved in the triggering of DNA fragmentation, an early event in apoptotic neuronal cell death after brain injury [175, 176]. *LARP4* gene product, the La-related protein 4, interacts with poly(A)-binding protein and promotes mRNA homeostasis [177]. *EIF1* codes for the eukaryotic initiation factor 1, which integrates the scanning mechanism of eukaryotic translation initiation [178]

Community D has only two hubs: *G3BP2*, which codes for a Ras-GTPase activating protein that contributes to stress granule formation following cellular stress [179, 180] and has its expression levels altered in TLE [181] and *FTSJ1*, whose gene product is a RNA methyltransferase expressed in the hippocampus and associated to intellectual disabilities and seizures [182]

The remaining communities containing high hierarchy genes in L-CO network are: B, which has the second largest number of nodes but harbors just one hub, *MLXIP* (aliase *MondoA*), which codes for a glucose sensing transcription factor involved in glucose homeostasis [183]; and G, containing two high-hubs, *TTL* that encodes a tubulin-tyrosine-ligase with a vital role in neuronal organization and control of neurite extensions [184], and *PPFIBP1* whose gene product is a liprin-family scaffold protein regulating cell adhesion, cell migration and synapse development [185].

The general picture of L-CO network is rather different of that depicted in E-CO, where the genes related to compensatory pathways predominated in the communities with strongest weight connections. Here the communities with the most relevant relationships harbor several genes related to pro-epileptic effects, seizure related mechanisms and vulnerability to epilepsy (*SLC6A1*, *S100B*, *RSCA1* and *PTPRZ1*), and just a few ones acting on compensatory mechanisms (*BMPR2) or* homeostasis (*MLXIP*). These different scenarios for CO networks will be discussed latter, considering the late and early onset forms of MTLE with a history of febrile seizures.

In both E- and L-CO networks the community with the largest number of nodes, i.e. community A (Fig 6C and 6D), was devoid of high-hierarchy genes, with most of their nodes well below of the *k*_*0*_ and *k*_*1*_ cut-off values (Fig 2B and 2D) adopted for these networks.

### Interactome network analysis

**Fig 7:**
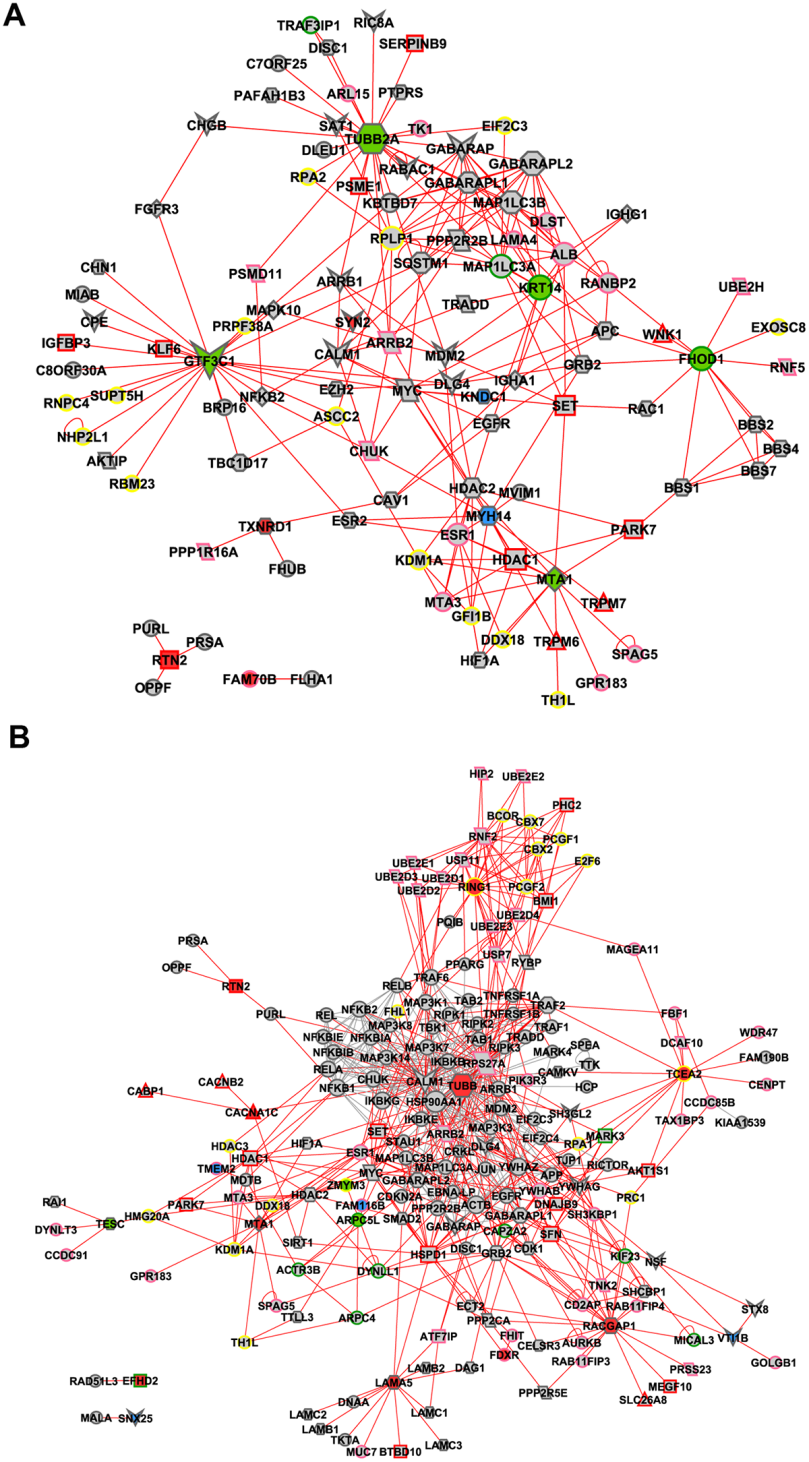
Interactome for DE networks. Interactome for E-DE (A) and L-DE (B) selected hubs (depicted in blue), VIPs (depicted in red) and high-hubs (depicted in green) using MINT and IntAct databases. Node size is related to node degree (number of links). Links in red represent the first and second node connections, centered in all hubs, VIPs and high-hubs for E-DE and in all hubs, VIPs and high-hubs, except TUBB for L-DE. Node shapes and border colors represent biological processes, as follows: parallelogram for apoptosis; parallelogram with pink border for ubiquitination; octagon for autophagy; circle with pink or green or yellow border stand for cell processes or cytoskeleton or transcriptional regulation respectively; diamond for inflammation; triangle with red border stand for ion channel; rectangle with green or red or pink stand for neurodegeneration or neuroprotection or response to oxidative stress respectively; hexagon for neuronal development; vee for synaptic transmission.

**Fig 8:**
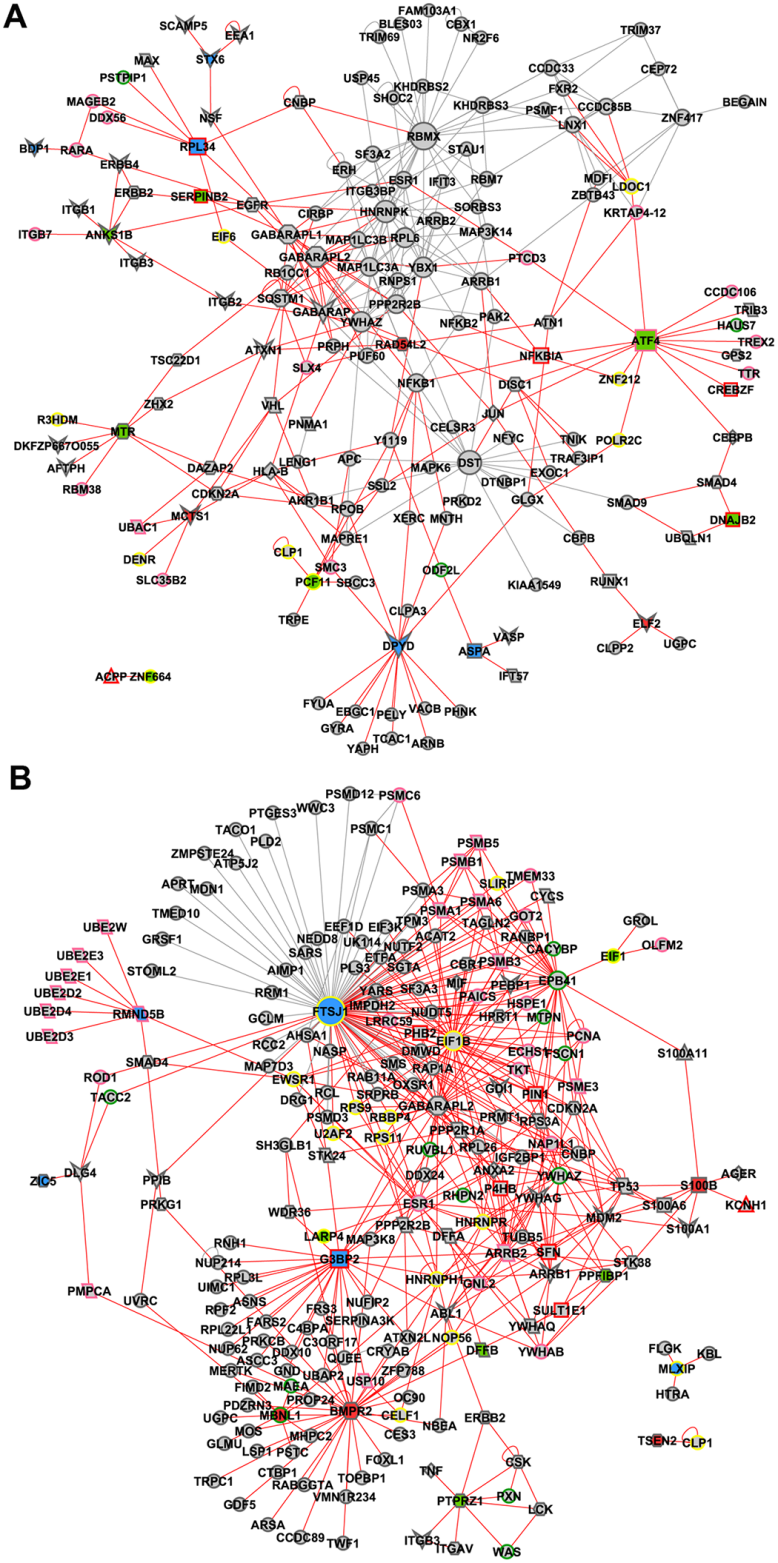
Interactome for CO networks. Interactome for E-CO (A) or L-CO (B) selected hubs (depicted in blue), VIPs (depicted in red) and high-hubs (depicted in green) using MINT and IntAct databases. Node size is related to node degree (number of links). Links in red represent the first and second node connections, for E-CO, or first, second and third node connections, for L-CO, centered in all hubs, VIPs and high-hubs, except FTSJ1 for L-CO. Node shapes and border colors represent biological processes, as follows: parallelogram for apoptosis; parallelogram with pink border for ubiquitination; octagon for autophagy; circle with pink or green or yellow border stand for cell processes or cytoskeleton or transcriptional regulation respectively; diamond for inflammation; triangle for ion binding; triangle with red border stand for ion channel; rectangle for neuroglia processes; rectangle with red or pink stand for neuroprotection or response to oxidative stress respectively; hexagon for neuronal development; vee for synaptic transmission.

An interactome analysis was performed in order to validate GCN results. Only the high hierarchy genes (hubs, VIPs or high-hubs) were considered in this analysis. MINT and IntAct databases were selected for data generation, which resulted in interactomes with 106 nodes and 222 edges for the E-DE group and 187 nodes and 690 edges for L-DE (Fig 7A and 7B); 161 nodes and 318 edges for E-CO and 215 nodes and 454 edges for L-CO (Fig 8A and 8B). The nodes (proteins) of interactomes corresponding to genes present in gene coexpression networks—and previously categorized as hubs, VIPs or high-hubs—appear colored in blue, red or green, respectively. Node size is related to node degree (number of links). Links in red represent the first and second node connections, centered in all hubs, VIPs and high-hubs, except for TUBB, a VIP in L-DE, and FTSJ1, a hub in L-CO, two ubiquitously distributed proteins. A functional description of the interactome nodes for all DE and CO networks based on Gene Ontology (biological process) and PubMed databases is presented in S1, S2, S3 and S4 Tables.

## Discussion

Community detection analysis of modular transcriptional repertoires for DE and CO gene coexpression networks, and the subsequent CGCS data analysis, revealed distinct molecular pathways for early- and late-onset forms of MTLE associated with childhood febrile seizures, as discussed below. Moreover, network connectivity is lower in both E networks when compared to the corresponding L networks, thus indicating that the hippocampal CA3 region of patients with early-onset FS-MTLE present a higher degree of dysregulation in cells’ functional organization [29, 32, 43]. Interactome analysis for experimentally verified protein-protein interactions confirmed the relevance of most high-hierarchy genes in E and L networks.

In the E-DE network coarse-grained community structure shows communities C and I as the ones with strongest connection weights. This C-I “axis”, described earlier in this paper, harbors high hierarchy genes with relevant roles in febrile-driven epilepsy and MTLE, such as *SCN9A*, *RTN2* and *BAIAP2*, in community C, and *B3GALTN1*, *KCN3* and *MTA1* in community I. This synaptic excitability “axis” is seconded by the axis A-B, whose high hierarchy genes are also related to pro-epileptic roles, as *SYN2* and *NIDD* (*ZDHHC23*). All this gene set is directly or indirectly involved with the molecular hallmarks of febrile seizures and epilepsy, namely: calcium and potassium channels, glutamate, NMDA receptors, HCN channels and HDACs [13]. Therefore on can conclude that E-DE network may well represent the “ground zero” of FS impact in hippocampal CA3.

The E-CO network, adopting the former analogy, would depict the derived “shock-waves” of FS impact on hippocampal CA3 cells. Indeed, the E-CO community with the strongest connection weights is community C, followed by communities D and H. The majority of the genes in the C-D-H interconnected communities are related to compensatory effects in epilepsy (seizure inhibition, neuronal survival and stress responses), what is in accordance with the concept that early onset epilepsies, although impacting more severely the ictal hippocampus, are associated to compensatory mechanisms [8]. Interestingly, this concept emerged from fMRI studies using network (graph theory) computational studies for assessing brain connectivity [6, 7, 8]. Hence, we can conclude that FS and FSE cause a perturbation in genomic and brain networks, determining “adaptive” rearrangements in both. The disease can be viewed as the breakdown of functional modules causing network reorganization. This issue will be further addressed below.

The L-DE network represents the “ground zero” of late-onset FS-MTLE, i.e., the GCN inferred from differentially expressed genes in the CA3 region of a hippocampus damaged by febrile IPI in early childhood but where temporal lobe epilepsy onset occurred after a long latent period (see Table 1). Here community C centers the strongest connection weights and also harbors most of the network’s VIPs and high hubs, what indicates its importance for network stability and functionality. The connection between C and F communities (Fig 6B) is the equivalent of the E-DE synaptic excitability C-I “axis”. Accordingly, the C-F “axis’ also harbors genetic and molecular hallmarks of epilepsy, such as: i) in community C the L-type calcium channel gene *CACNA1*, *RTN2*, a regulator of EAAC1 (also a VIP in E-DE network), the calcium binding/signaling genes *TESC*, *CALM3*, *EFHD2*; ii) in community F the gene *CTNAP1*, regulator of the glutamate receptor subunit GluA1. Moreover, as described before, other significant connections of community C—the communities A, H, and B—include several genes involved in epilepsy processes, like *SXN25* and *ARPC5L*, markers of intractable epilepsy. Since L-DE high hierarchy genes, as a whole, are involved neuronal excitability or playing roles in other epilepsy-related processes, this network is functionally similar to E-DE. However, it reflects distinctive adaptive response to febrile seizures, probably related to late-onset MTLE development.

Contrarily to the observed for E-CO network, L-CO communities with strongest weighted connections harbor more high hierarchy genes related to pro-epileptic effects, or vulnerability to epilepsy, than to compensatory mechanisms. This finding, fully described in the Results section, is in accordance to the concept that early-onset epilepsies are associated with compensatory mechanisms, because the younger brain is more plastic, whereas in late-onset developed epilepsies the mature injured brain is less able to generate adaptive compensatory mechanisms [8, 186].

Imaging studies (structural and functional MRI) and graph theory models of brain connectivity led to significant progress in understanding the pathophysiology of temporal lobe epilepsy, [6, 8, 187]. Essentially, these studies showed that TLE is a network disease, i.e. a system disorder that alters local and distributed brain networks [6, 188]. Similarly to the application of complex network analysis to the study of gene-gene and protein-protein interactions, graph theory modeling of brain networks allows a quantitative description of the topological organization of brain connectivity based on network property measures, where nodes represent brain regions interconnected by edges. In this context, communities, or modules, are groups of highly connected nodes within the brain networks, modularity describes hierarchical organization, and network hubs are the nodes with greater degree centrality, i.e., those mediating most of the short path lengths between nodes. According to their community insertion and connectivity profile, hubs mediate intra- or inter module connectivity [6, 26, 188].

Complex network analyses of human brain connectivity revealed that the hippocampal formation has a concentration of densely linked nodes with very high degree centrality [189]. Increased clustering, an indicator of low connectivity, and alterations in the distribution of network hubs were detected in patients with refractory MTLE comparatively to normal controls [190]. Moreover, it was found, by comparing brain connectivity parameters of early- and late-onset MTLE patients who underwent resting-state fMRI scan, that late-onset patients had lower connectivity and higher modularity [8], thus indicating a certain degree of modular interaction disorganization in late-onset cases. These MRI data have a striking similarity with our findings on community structure analysis of transcriptional networks in early- and late-onset MTLE: i) comparative alterations in modularity/connectivity and hub and modular distributions; ii) evidences that in late MTLE development the brain is less able to generate adaptive mechanisms.

Altogether, gene coexpression and MRI networks studies on MTLE show that this disease, as other chronic non-communicable diseases, stems from environmentally-induced perturbations of complex intra and intercellular networks, probably modulated by the individual’s genomic makeup. Network reorganization following these perturbations may be kept stable for long periods due to epigenetic mechanisms acting just after the insult, and/or just after epilepsy onset [18, 19]. In fact, it was shown in kainic acid mouse model of epilepsy that genome-wide methylation status change after status epilepticus and in epileptic tolerance [191], thus contributing to regulate gene expression (and to reorganize gene-gene interaction networks) in the seizure-damaged hippocampus.

The complex network analyses performed in this study allowed a broad and more detailed view of genomic and molecular mechanisms involved in early and late-onset MTLE, in comparison to analyses centered solely on differentially expressed genes. Network centrality in DE and CO networks, is consistent with the network disease model, where a group of nodes whose perturbation (e.g. febrile IPI) leads to a disease phenotype occupies a central position in the network [4, 28, 192].

Most importantly, our data on hippocampal CA3 gene coexpression networks are in agreement with previous fMRI data showing that early onset epilepsies, although impacting more severely the ictal hippocampus, are associated to compensatory mechanisms, while in late MTLE development the brain is less able to generate adaptive mechanisms. This is very significant if one considers that the identification of windows of opportunity for antiepileptogenic interventions depends on a better understanding of the mechanisms occurring during the seizure-free interval, or latent period, in MTLE [23]. On the other hand, the probability of exerting therapeutic effects through the modulation of particular genes will be higher if these genes are highly interconnected in transcriptional networks [4, 2, 193]. In epilepsy—a disease affecting more than 50 million people around the world and where 30% of the patients do not respond to the available antiepileptic drugs—this systems biology approach seems to be mandatory for discovering new multi-target drugs, since hitting a single target does not treat complex diseases. In conclusion, a network-based approach to intractable epilepsy would be probably more effective than the “silver bullets” sought at the beginning of medical genomics.

## Supporting Information

S1 Fig Epilepsy duration and age at surgery. Scatter-plot of epilepsy duration (**Figure A**) and age at surgery (**Figure B**), in years, for early and late-onset MTLE patients and t-test p-value.
(TIF)

S2 Fig Node distribution and categorization for F community in the L-DE network. Scatter plots of node degree (*k*0) vs concentric node degree (*k*1) measures of GO annotated genes in L-DE. Hubs (blue), VIPs (red) and high-hubs (green), identified by their gene symbols. The nodes from community F are identified by orange dots/gene symbols.
(TIF)

S1 Table Early DE interactome. Functional description of interactome nodes linked in first and second levels; centered in hubs, high-hubs and VIPs.
(PDF)

S2 Table Late DE interactome. Functional description of interactome nodes linked in first and second levels; centered in hubs, high-hubs and VIPs.
(PDF)

S3 Table Early CO interactome. Functional description of interactome nodes linked in first and second levels; centered in hubs, high-hubs and VIPs.
(PDF)

S4 Table Late CO interactome. Functional description of interactome nodes linked in first and second levels; centered in hubs, high-hubs and VIPs.
(PDF)

S1 Video Complete transcriptional interaction network for early-onset MTLE based on Pearson’s correlation of 9,578 GO annotated genes. High-hubs, Hubs and VIPs are identified by their gene symbols. Communities are indicated by different colors.
(MP4)

S2 Video Complete transcriptional interaction network for late-onset MTLE based on Pearson’s correlation of 11,321 GO annotated genes. High-hubs, Hubs and VIPs are identified by their gene symbols. Communities are indicated by different colors.
(MP4)
