# Supplementary figures and images for: Community Structure Analysis of Transcriptional Networks Reveals Distinct Molecular Pathways for Early- and Late-Onset Temporal Lobe Epilepsy with Childhood Febrile Seizures
Source: PLoS One. 2015 May 26;10(5):e0128174. doi: 10.1371/journal.pone.0128174 (PMC4444281; doi:10.1371/journal.pone.0128174)

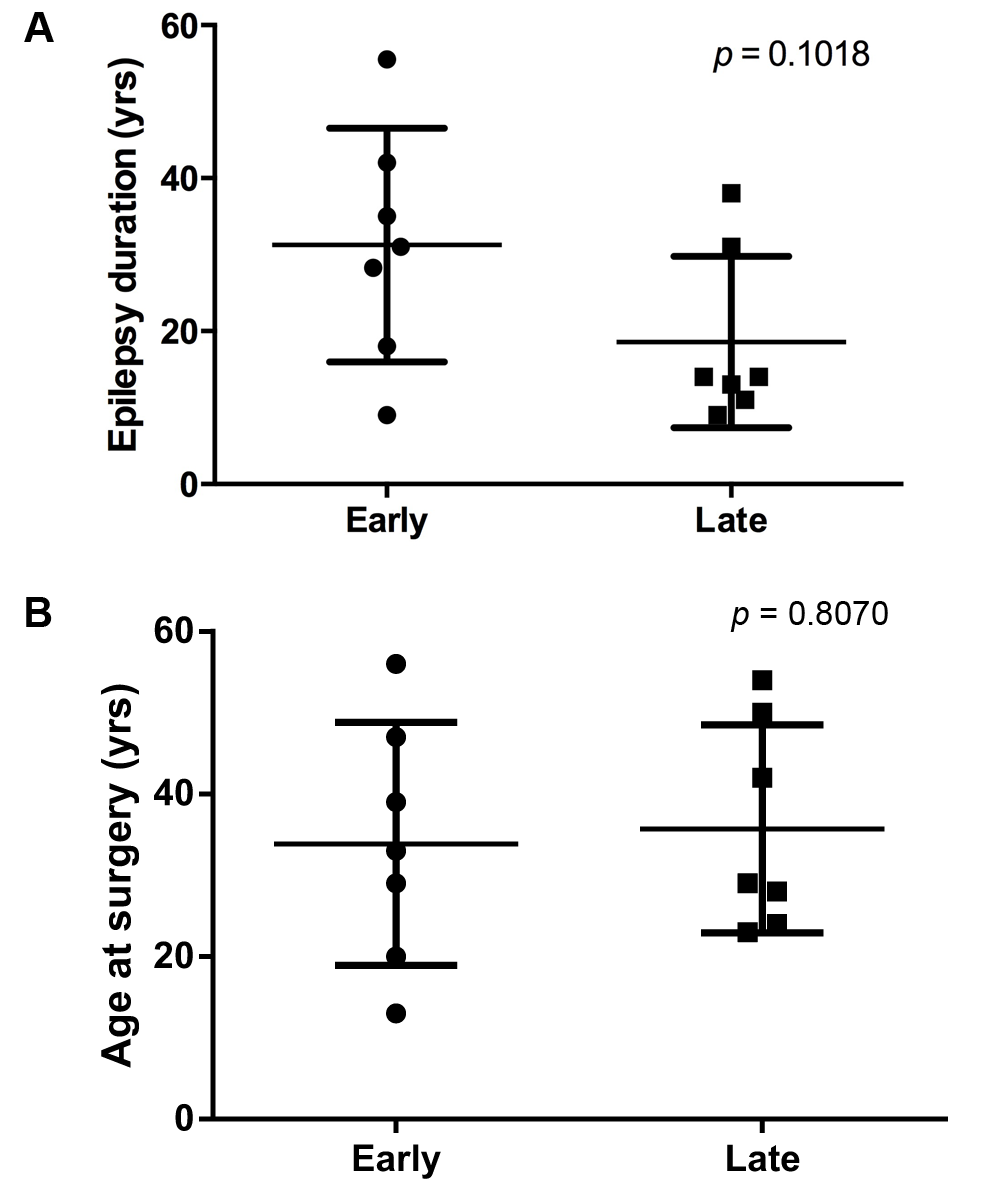

Supplement: S1 Fig — Scatter-plot of epilepsy duration (Figure A) and age at surgery (Figure B), in years, for early and late-onset MTLE patients and t-test p-value. (TIF) [file pone.0128174.s001.tif]

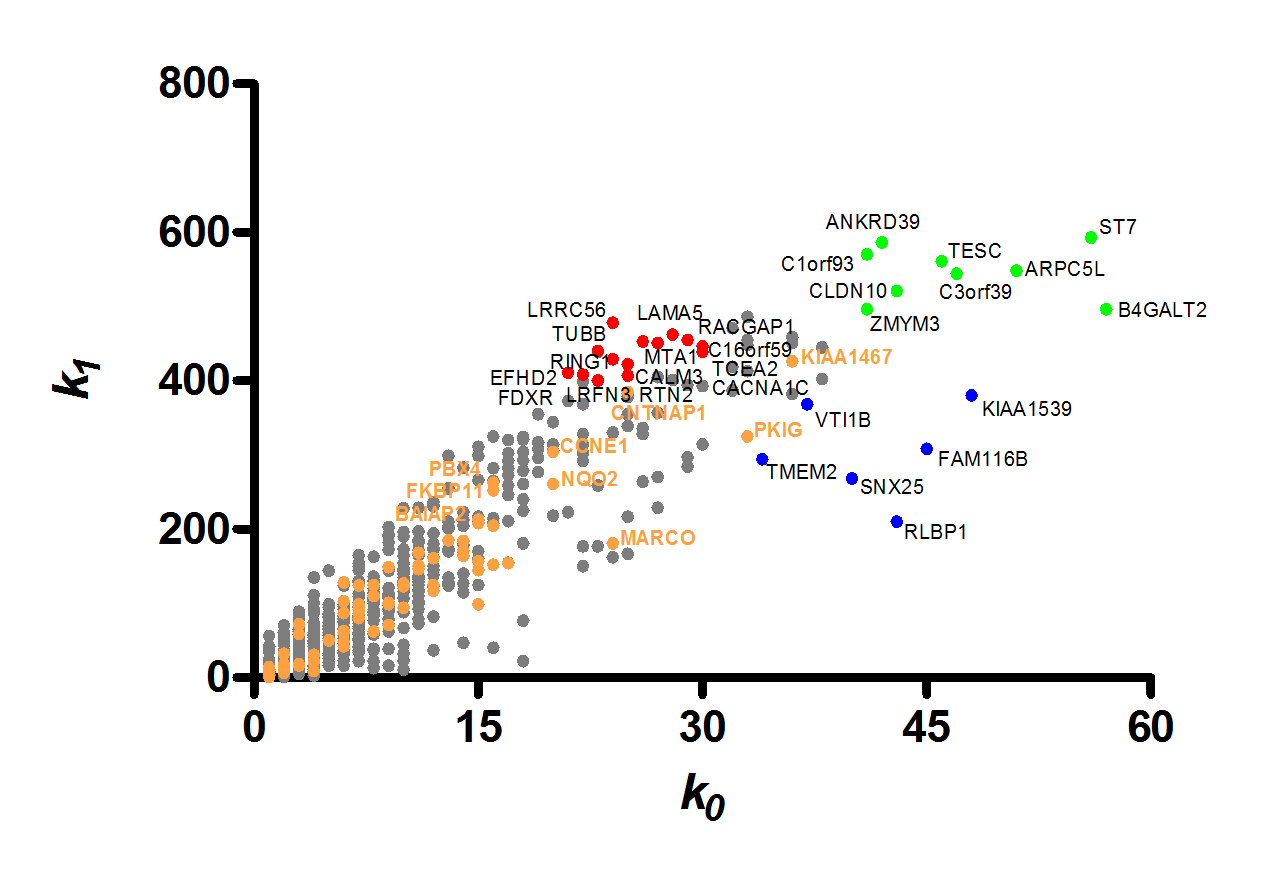

Supplement: S2 Fig — Scatter plots of node degree (k0) vs concentric node degree (k1) measures of GO annotated genes in L-DE. Hubs (blue), VIPs (red) and high-hubs (green), identified by their gene symbols. The nodes from community F are identified by orange dots/gene symbols. (TIF) [file pone.0128174.s002.tif]
