# Supplementary material for: Community Structure Analysis of Transcriptional Networks Reveals Distinct Molecular Pathways for Early- and Late-Onset Temporal Lobe Epilepsy with Childhood Febrile Seizures
Source: PLoS One. 2015 May 26;10(5):e0128174. doi: 10.1371/journal.pone.0128174 (PMC4444281; doi:10.1371/journal.pone.0128174)
Supplement: S1 Table — Functional description of interactome nodes linked in first and second levels; centered in hubs, high-hubs and VIPs. (PDF) [file pone.0128174.s003.pdf]

**Table S1. Early DE interactome network**. Functional description of interactome nodes linked in first and second levels - centered in hubs, high-hubs and VIPs.

| Protein | PubMed | Biological Process | Shape (border color) |
|---|---|---|---|
| AKTIP | apoptotic process | apoptosis | parallelogram |
| MYC | cell cycle progression; apoptosis; cellular transformation | | |
| PPP2R2B | apoptotic process | | |
| TRADD | induction of apoptosis by extracellular signals; signal transduction | | |
| MAP1LC3B | autophagy/microtubule assembly | autophagy | octagon |
| SQSTM1 | autophagy; apoptotic process; endosomal transport | | |
| TBC1D17 | Rab GTPase activator activity | | |
| ALB | Albumin (ALB) functions as a carrier protein for steroids, fatty acids, and thyroid hormones; plays a role in stabilizing extracellular fluid volume | cell processes | circle (pink) |
| FAM70B | Official symbol: TMEM255B. Membrane component | | |
| SPAG5 | cytoplasmic trafficking; spindle organization; cilium formation | | |
| TK1 | nucleotide metabolism | | |
| DLST | glucose metabolism | cell processes/ bioenergetic systems | circle (pink) |
| ESR1 | estrogen receptor alpha/regulator of energy balance | | |
| RANBP2 | regulation of glucose transport | | |
| LAMA4 | regulation of cell adhesion; regulation of cell migration | cell processes/cell-cell interaction/adhesion | circle (pink) |
| MTA3 | zinc ion binding; regulation of cell proliferation | cell processes/proliferation | circle (pink) |
| ARL15 | small GTPase mediated signal transduction | cell processes/signaling | circle (pink) |
| GPR183 | G protein-coupled receptor; signaling | | |
| KRT14 | Cell proliferation and differentiation | cytoskeleton | circle (green) |
| TRAF3IP1 | cytoskeletal system protein | | |
| FHOD1 | actin binding; positive regulation of stress fiber assembly | cytoskeleton/actin | circle (green) |
| MAP1LC3A | microtubule-associated protein | cytoskeleton/tubulin | circle (green) |
| FGFR3 | JAK-STAT cascade | inflammation/ immune function | diamond |
| IGHA1 | immune response | | |
| IGHG1 | immune response | | |
| MAPK10 | JUN kinase activity; activation of MAPK activity | | |
| NFKB2 | inflammation and immune function | | |
| MTA1 | ubiquitination and acetylation; inflammatory response | inflammation/ubiquitination | diamond |
| TRPM6 | calcium channel activity | ion channel/voltage-gated channel | triangle (red) |
| TRPM7 | calcium channel activity | | |
| WNK1 | ATP binding; sodium and chloride ion transport regulator | | |
| BBS1 | nonmotile primary cilium assembly | neuronal development | hexagon |
| BBS4 | alpha-tubulin binding; brain morphogenesis; cerebral cortex development | | |
| BBS7 | Recruits Rab8(GTP) to the primary cilium promoting ciliogenesis | | |
| PAFAH1B3 | brain development; nervous system development; lipid metabolic process | | |
| TXNRD1 | Brain development; expressed in glia and neuron precursor cells | | |
| APC | multisubunit E3 ubiquitin ligase controlling cell cycle transition in proliferating cells | neuronal development/plasticity | hexagon |
| CAV1 | cholesterol transport; neuronal cholesterol homeostasis | | |
| CHN1 | GTPase activator activity; regulation of axonogenesis | | |
| ESR2 | estrogen receptor beta | | |
| TUBB2A | neuron differentiation; learning and memory processes | | |
| DISC1 | microtubule cytoskeleton organization; neuron migration | neuronal development/plasticity/cell migration | hexagon |
| EGFR | cell proliferation; axon guidance; cerebral cortex cell migration; interaction of EGFR and EMP-1 plays a role in the mechanism of drug resistance in epilepsy | | |
| HIF1A | cell differentiation; neural crest cell migration | | |
| RAC1 | axon guidance; cell adhesion; cell motility | | |
| GRB2 | axon elongation; adaptor molecule in several growth factor signaling cascades; association with DISC1 | neuronal development/plasticity/dendritic growth | hexagon |
| HDAC2 | dendrite development; negative regulation of neuron projection development | | |
| KNDC1 | dendrite growth and morphogenesis | | |
| MYH14 | neuritogenesis | | |
| BBS2 | brain development; cerebral cortex development; hippocampus development | neuronal development/plasticity/hippocampus | hexagon |
| EZH2 | methyltransferase/neurogenesis and memory | | |
| PTPRS | cell adhesion; developmental of cerebellum, cerebral cortex, corpus callosum, hippocampus and spinal | | |
| HDAC1 | histone deacetylase activity; negative regulation of apoptosis | neuroprotection | rectangle (red) |
| IGFBP3 | regulation of apoptotic process | | |
| KLF6 | zinc ion binding; positive regulation of transcription | | |
| PARK7 | protects neurons against oxidative stress and cell death; regulation of neuron apoptotic process; membrane depolarization | | |
| PSME1 | proteasome activator | | |
| RTN2 | neuroprotection; Intracellular vesicular transport; glucose import | | |
| SERPINB9 | anti-apoptosis | | |
| SET | negative regulation of neuron apoptosis | | |
| ARRB1 | G-protein coupled receptor internalization; positive regulation of Rho protein signal transduction | synaptic transmission | vee |
| CALM1 | calcium-binding; signal transduction | | |
| CHGB | large dense core vesicle component | | |
| CPE | peptide hormones/neurotransmitters biosynthesis | | |
| DLG4 | protein localization to synapse; axon guidance; nervous system development | | |
| GTF3C1 | synaptic activation; control of dendritic branching | | |
| MDM2 | ubiquitin-protein ligase activity; zinc ion binding; synaptic transmission | | |
| RABAC1 | proline-rich region binding; synaptic vesicle | | |
| RIC8A | G-protein alpha-subunit binding | | |
| SYN2 | regulation of epileptic and synaptic activity/plasticity on hippocampus | | |
| GABARAP | GABA receptor binding; synaptic transmission | synaptic transmission/GABA | vee |
| SAT1 | glutamine transporter | | |
| GABARAPL1 | GABA receptor binding; autophagy | synaptic transmission/GABA/autophagy | vee |
| GABARAPL2 | GABA receptor binding; autophagy; intra-Golgi vesicle-mediated transport; protein transport | | |
| ASCC2 | regulation of transcription | transcriptional regulation | circle (yellow) |
| DDX18 | member of the DEAD box protein family; alteration of RNA secondary structure; ATPase activity | | |

| | | | |
|---|---|---|---|
| EIF2C3 | RNA binding; negative regulation of translation involved in gene silencing by miRNA | | |
| EXOSC8 | rRNA processing | | |
| GFI1B | zinc ion binding; negative regulation of histone H3-K4 methylation | | |
| NHP2L1 | snoRNA binding; RNA splicing | | |
| PRPF38A | RNA splicing | | |
| RBM23 | mRNA processing | | |
| RNPC4 | Official Symbol: RBM23. RNA binding protein; mRNA processing | | |
| RPA2 | transcription-coupled nucleotide-excision repair | | |
| RPLP1 | RNA metabolic process; translation | | |
| SUPT5H | negative regulation of transcription elongation | | |
| TH1L | negative regulation of transcription | | |
| KDM1A | histone demethylase activity (H3-K4 specific); chromatin modification | transcriptional regulation/neurite morphogenesis | circle (yellow) |
| CHUK | inhibitor of NF-kappa-B complex | ubiquination | parallelogram (pink) |
| ARRB2 | G-protein coupled receptor binding; ubiquitin protein ligase binding | | |
| PPP1R16A | protein phosphatase 1 | | |
| PSMD11 | protein polyubiquitination | | |
| UBE2H | ubiquitin-protein ligase activity | | |
| RNF5 | RING-finger/control of cell motility/ubiquitination | | |
