# Supplementary material for: Community Structure Analysis of Transcriptional Networks Reveals Distinct Molecular Pathways for Early- and Late-Onset Temporal Lobe Epilepsy with Childhood Febrile Seizures
Source: PLoS One. 2015 May 26;10(5):e0128174. doi: 10.1371/journal.pone.0128174 (PMC4444281; doi:10.1371/journal.pone.0128174)
Supplement: S2 Table — Functional description of interactome nodes linked in first and second levels; centered in hubs, high-hubs and VIPs. (PDF) [file pone.0128174.s004.pdf]

Table S2. Late DE interactome network. Functional description of interactome nodes linked in first and second levels - centered in hubs, high-hubs and VIPs.

Protein	PubMed	Biological Process	Shape (border color)
MYC	cell cycle progression; apoptosis; cellular transformation	apoptosis	parallelogram
PPP2CA	induction of apoptosis; mesoderm development; regulation of Wnt receptor signaling pathway		
PPP2R5E	apoptotic process		
RYBP	apoptotic process; histone H2A monoubiquitination; multicellular organismal development		
TRAF2	autophagy	autophagy	octagon
AURKB	serine/threonine kinase	cell processes	circle (pink)
CENPT	centromere assembly		
FHIT	nucleic acid metabolism		
GOLGB1	Golgi organization		
MAGEA11	melanoma antigen family A; primate-specific steroid receptor coregulator		
MUC7	mucin peptide; O-glycan processing; cellular protein metabolic process		
RAB11FIP4	endosomal recycling regulation		
SPAG5	cytoplasmic trafficking; spindle organization; cilium formation		
WDR47	multicellular organismal development		
ESR1	estrogen receptor alpha/regulator of energy balance	cell processes/bioenergetic systems	circle (pink)
TMEM2	cell movement and tissue morphogenesis	cell processes/cell migration	circle (pink)
FDXR	Iron–sulfur cluster biogenesis; maintenance of cytosolic and mitochondrial iron homeostasis	cell processes/iron homeostasis	circle (pink)
SH3KBP1	apoptosis; cytoskeletal rearrangement; cell adhesion; regulation of clathrin-dependent endocytosis	cell processes/neuronal migration	circle (pink)
MTA3	zinc ion binding; regulation of cell proliferation	cell processes/proliferation	circle (pink)
CCDC85B	cell differentiation; cell growth	cell processes/proliferation/differentiation	circle (pink)
CD2AP	signal transduction; cell migration; cell extension; cell cycle		
DYNLT3	protein binding; cell cycle		
TAX1BP3	signal transduction; cell proliferation		
FBF1	protein binding	cell processes/protein binding	circle (pink)
CCDC91	g-protein signaling in the development and maintenance of white matter microstructure	cell processes/signaling	circle (pink)
GPR183	G protein-coupled receptor; signaling		
PIK3R3	regulation of phosphorylation		
RAB11FIP3	endosomal trafficking during cytokinesis		
YWHAB	signal transduction/14-3-3 protein family		
FAM116B	Official symbol: DENND6B; Rab-mediated trafficking pathway	cell processes/transport	circle (pink)
ACTR3B	actin binding; cell motility	cytoskeleton/actin	circle (green)
ARPC4	actin filament binding; cell motility		
ARPC5L	regulation of actin filament polymerization		
CAPZA2	actin cytoskeleton organization		
MICAL3	regulation of actin microfilaments		
DYNLL1	intracellular transport and motility	cytoskeleton/cell motility	circle (green)
KIF23	mitotic spindle elongation; microtubule movement	cytoskeleton/tubulin	circle (green)
MTA1	ubiquitination and acetylation; inflammatory response	inflammation/ubiquitination	diamond
CABP1	regulation of voltage-gated calcium channel activity	ion channel/voltage-gated channel	triangle (red)
CACNA1C	Calcium channel; learning; memory and brain plasticity; disturbance of long-term potentiation		
CACNB2	voltage-dependent calcium channel proteincalcium channel activity; neuromuscular junction development		
SLC26A8	anion transport; chloride transport		
MARK3	phosphorylation of tau proteins MAP2 and MAP4	neurodegeneration	retangle (green)
EFHD2	Conserved calcium-binding protein, abundant within the central nervous system; EFhd2 may play an important role in the pathobiology of tau-mediated neurodegeneration	neurodegeneration/ion-binding/Ca+2 binding	retangle (green)
CAMKV	calmodulin-binding, vesicle-associated protein kinase in axons and dendrites	neuronal development	hexagon
CRKL	activate the RAS and JUN kinase signaling pathways; regulation of postnatal hippocampal dendritogenesis		
DAG1	microtubule anchoring; dual receptor for agrin and laminin-2 in the Schwann cell membrane; dendrite orientation; neuronal migration	neuronal development/plasticity	hexagon
ECT2	nervous system development; positive regulation of neuron differentiation; apoptotic process; cell cycle, differentiation and morphogenesis;		
RAI1	induced by retinoic acid; neuron development; learnig and memory		
SHCBP1	regulation of neural precursor cell proliferation		
TESC	EF-hand protein with a single Ca2+-binding site; interacts with calcineurin		
TTLL3	axoneme assembly; cilium assembly		
TUBB	neuronal differentiation; cell viability; cytoskeleton structure		
HIF1A	cell differentiation; neural crest cell migration	neuronal development/plasticity/cell migration	hexagon
RACGAP1	neuronal proliferation; cell migration; cytoskeletal reorganization; cell cycle regulation		
CDK1	regulation of Schwann cell differentiation; cell cycle control; neurite outgrowth	neuronal development/plasticity/dendritic growth	hexagon
CELSR3	calcium ion binding; neuron migration; neuropeptide signaling pathway; axonal fasciculation		
GRB2	axon elongation; adaptor molecule in several growth factor signaling cascades; association with DISC1		
HDAC2	dendrite development; negative regulation of neuron projection development		
LAMA5	Matrix glycoprotein; cell adhesion; differentiation; migration; signaling; neurite outgrowth		
LAMB1	cell adhesion; differentiation; migration; signaling; neurite outgrowth		
LAMB2	cell adhesion; differentiation; migration; signaling; neurite outgrowth		
LAMC1	cell adhesion; differentiation; migration; signaling; neurite outgrowth; axon myelination		
LAMC2	cell adhesion; differentiation; migration; signaling; neurite outgrowth		
LAMC3	cell adhesion; differentiation; migration; signaling; neurite outgrowth		
SIRT1	axonal elongation, neurite outgrowth, dendritic branching		
SMAD2	dendritic growth and complexity and neuron cell body size		
AKT1S1	neuroprotection	neuroprotection	rectangle (red)

BMI1	Polycomb transcriptional repressor, is implicated in cell cycle regulation and cell senescence.		
BTBD10	Akt interactor; regulation of neuron survival		
DNAJB9	protection of stressed cells from apoptosis		
HDAC1	histone deacetylase activity; negative regulation of apoptosis		
HSPD1	chaperone-mediated protein complex assembly		
MEGF10	cell adhesion; cell projection; mediator of astrocyte phagocytosis		
PARK7	protects neurons against oxidative stress and cell death; regulation of neuron apoptotic process; membrane depolarization		
PHC2	neuronal survival		
RTN2	neuroprotection; Intracellular vesicular transport; glucose import		
SET	negative regulation of neuron apoptosis		
SFN	signal transduction/14-3-3 protein family		
ATF7IP	control of hippocampal GABABR1a and GABABR1b subunit gene expression; resposnse to oxidative stress	response to oxidative stress	rectangle (pink)
CALM1	calcium-binding; signal transduction	synaptic transmission	vee
DLG4	protein localization to synapse; axon guidance; nervous system development		
SH3GL2	synaptic vesicle endocytosis; axon guidance; central nervous system development		
YWHAG	14-3-3 protein; regulation of neuron differentiation; regulation of synaptic plasticity		
NSF	GABAB signaling efficacy; synaptic transmission; exocytosis; membrane fusion events; dendritic shaft	synaptic transmission/GABA	vee
GABARAP	GABA receptor binding; synaptic transmission		
GABARAPL1	GABA receptor binding; autophagy	synaptic transmission/GABA/autophagy	vee
GABARAPL2	GABA receptor binding; autophagy; intra-Golgi vesicle-mediated transport; protein transport		
STX8	vesicle transport and fusion (t-SNARE family); neurite outgrowth	synaptic transmission/SNARE complex	vee
VTI1B	Neuronal development; axon morphology; endosomal membrane trafficking (SNARE complex)		
SNX25	TGF-beta signaling; development of temporal lobe epilepsy	synaptic transmission/TLE	vee
BCOR	heat shock protein binding; chromatin modification	transcriptional regulation	circle (yellow)
CBX2	chromatin remodeling and modification of histones; regulation of transcription		
CBX7	regulation of transcription		
DDX18	member of the DEAD box protein family; alteration of RNA secondary structure; ATPase activity		
E2F6	transcription factor; cell cycle control		
FHL1	zinc finger protein		
HDAC3	histone deacetylation; chromatin modification		
HMG20A	regulation of transcription, DNA-dependent		
PCGF1	histone H2A monoubiquitination; regulation of transcription		
PCGF2	negative regulator of transcription (Polycomb group)		
PRC1	regulation of transcription (Polycomb group)		
RING1	Transcriptional repressor/activator (Polycomb group); metal ion binding		
RPA1	transcription-coupled nucleotide-excision repair		
TCEA2	Aliase: TFIIS. SII class transcription elongation factor		
TH1L	negative regulation of transcription		
ZMYM3	gene silencing; zinc ion binding;		
KDM1A	histone demethylase activity (H3-K4 specific); chromatin modification	transcriptional regulation/neurite morphogenesis	circle (yellow)
RNF2	RING-finger/control of cell motility/ubiquitination	ubiquitination	parallelogram (pink)
RPS27A	ubiquitin pathway		
ARRB2	G-protein coupled receptor binding; ubiquitin protein ligase binding		
DCAF10	protein ubiquitination		
HIP2	Official Symbol: UBE2K. Suppression of apoptosis; ubiquitin-dependent protein catabolic process		
PRSS23	proteolysis		
TNK2	protein ubiquitination; regulation of clathrin-mediated endocytosis; signal transduction		
UBE2D1	positive regulation of protein ubiquitination; cell cycle		
UBE2D2	protein binding; ubiquitination		
UBE2D3	apoptotic process; protein ubiquitination		
UBE2D4	protein ubiquitination		
UBE2E1	cell cycle; ubiquitination		
UBE2E2	protein ubiquitination		
UBE2E3	protein ubiquitination; regulation of growth		
USP11	protein ubiquitination; proteolysis		
USP7	protein deubiquitination; apoptosis; multicellular organismal development;		
