# Supplementary material for: Community Structure Analysis of Transcriptional Networks Reveals Distinct Molecular Pathways for Early- and Late-Onset Temporal Lobe Epilepsy with Childhood Febrile Seizures
Source: PLoS One. 2015 May 26;10(5):e0128174. doi: 10.1371/journal.pone.0128174 (PMC4444281; doi:10.1371/journal.pone.0128174)
Supplement: S3 Table — Functional description of interactome nodes linked in first and second levels; centered in hubs, high-hubs and VIPs. (PDF) [file pone.0128174.s005.pdf]

Table S3. Early CO interactome network. Functional description of interactome nodes linked in first and second levels - centered in hubs, high-hubs and VIPs.

Protein	PubMed	Biological Process	Shape (border color)
IFT57	aliase: HIPPI. Apoptotic process; intrafagellar transport	apoptosis	parallelogram
MAX	MYC associated factor X. Involved in cell proliferation, differentiation; apoptosis		
PNMA1	neuron- and testis-specific protein; proapoptotic protein in neurons		
TRIB3	negative regulator of NF-kappaB; can also sensitize cells to TNF- and TRAIL-induced apoptosis		
VHL	neuronal differentiation; neuronal apoptosis		
UBQLN1	apoptotic process; protein ubiquitination	apoptosis/ubiquitination	parallelogram
SQSTM1	autophagy; apoptotic process; endosomal transport	autophagy	octagon
CCDC106	cell differentiation; cell growth	cell processes	circle (pink)
DDX56	member of the DEAD box protein family; alteration of RNA secondary structure; ATPase activity		
KRTAP4-12	keratin-associated protein		
MAGEB2	cell growth control; epigenetic reprogrammed by valproic acid		
PTCD3	component of mitochondrion; mitochondrial translation		
RARA	regulation of development, differentiation, apoptosis		
RBM38	Aliase: RNPC1. Cell differentiation; cell cycle		
SLX4	DNA repair and recombination		
SMC3	component of the multimeric cohesin complex; chromosome segregation; regulator of RUNX1		
TREX2	double-stranded DNA break repair		
ITGB7	cell signaling; cell adhesion	cell processes/cell-cell interaction/adhesion	circle (pink)
SLC35B2	solute carrier; transmembrane transport	cell processes/transport	circle (pink)
TTR	carrier protein for thyroid hormones, cerebrospinal fluid and retinol transport		
HAUS7	centrosome and mitotic spindle integrity	cytoskeleton	circle (green)
ODF2L	component of cytoskeleton; formation of mother centriole distal/subdistal appendages and generation of primary cilia		
PSTPIP1	scaffold protein and regulator of the actin cytoskeleton	cytoskeleton/actin	circle (green)
JUN	JUN (aliase AP-1) activation is associated with increased neuronal cell death	inflammation/cell death	diamond
CEBPB	regulation of genes involved in immune and inflammatory responses	inflammation/immune function	diamond
HLA-B	antigen processing and presentation via MHC class I; immune response		
ACPP	general regulatory partner of Shaw-like K(+) channels (KCNC); acid phosphatase activity	ion channel/voltage-gated channel	triangle (red)
RUNX1	neuronal differentiation and microglial activation	neuroglia processes	rectangle
ASPA	oligodendrocyte maturation and myelination; glutamatergic synaptic activity	neuroglia processes/oligodendrocyte differentiation	rectangle
CNBP	Aliases: ZNF9 and DM2. Zinc finger protein; sterol-mediated transcriptional regulation; brain development	neuronal development	hexagon
MTR	metabolism of homocysteine, which is involved in epileptogenesis; nervous system development		
ATN1	central nervous system development; neuron apoptosis; regulation of neuron differentiation	neuronal development/plasticity	hexagon
DAZAP2	cell signaling and transcription regulation; Wnt/beta-catenin-signalling pathway; posterior neural fate; essential branch of FGF-induced neural patterning		
ERBB2	nervous system development; glial cell differentiation; axon guidance		
GPS2	interacts with NR3A, linking NMDA receptor activation to suppression of RAS/MAPK-mediated signaling; cell survival; p53 activity modulation		
RAD54L2	aliase ARIP4. Neuronal development		
TSC22D1	cell proliferation, differentiation and apoptosis		
ZHX2	negative regulation of neuron differentiation; zinc finger protein		
DISC1	microtubule cytoskeleton organization; neuron migration	neuronal development/plasticity/cell migration	hexagon
EGFR	cell proliferation; axon guidance; cerebral cortex cell migration; interaction of EGFR and EMP-1 plays a role in the mechanism of drug resistance in epilepsy		
CDKN2A	Aliases: p16; INK4A. cell cycle; epigenetic response during nerve regeneration; axonal lenght	neuronal development/plasticity/dendritic growth	hexagon
SMAD4	dendritic growth and complexity and neuron cell body size		
CREBZF	Zhangfei; potential regulator of unfolded protein response	neuroprotection	rectangle (red)
DNAJB2	neuroprotection; regulation of cell growth; protein ubiquitination		
NFKBIA	member of the NF-kappa-B inhibitor family; inflammatory responses; neurite outgrowth and cell survival		
RPL34	regulation of CDK5, a mediator of neuronal death and survival		
SERPINB2	neuroprotection induced by synaptic activity		
ATF4	control of hippocampal GABABR1a and GABABR1b subunit gene expression; response to oxidative stress	response to oxidative stress	rectangle (pink)
AFTPH	clathrin-mediated trafficking in neurons	synaptic transmission	vee
ANKS1B	postsynaptic signaling scaffolding protein; cellular component of Cajal body; postsynaptic density and postsynaptic membrane; ephrin receptor binding		
ATXN1	regulation of excitatory postsynaptic membrane potential		
BDP1	Rearrangement of nuclear architecture; interacts with TFIIC for synaptic activation and dendritic length and branching control		
DKFZP667O055	official name: CRELD2. Interacts with cytoplasmic domain of human neuronal nicotinic acetylcholine receptor; ER stress response; calcium ion binding		
DPYD	neurotransmission (b-alanine) modulation; electron carrier activity		
EEA1	endosomal trafficking; neurite outgrowth; synaptic plasticity; recycling of synaptic vesicles and neurotransmitter receptors		
ELF2	regulation of neurotransmission during endoplasmic reticulum stress		
ITGB1	cell adhesion; synaptic development; axonal outgrowth		
ITGB2	synaptic contacts and synaptogenesis of hippocampal neurons		
ITGB3	cell adhesion; migration; signaling; modulator of serotonergic systems		
VASP	spine modulation and synapse formation and maturation; spine head enlargement; axon guidance; synaptic plasticity		
ERBB4	morphological and functional development of excitatory synapses in GABAergic neurons	synaptic transmission/GABA	vee
MCTS1	carboxylate transporter involved in TLE by influencing brain energy homeostasis; mitochondrial function, GABA-ergic and glutamatergic neurotransmission; flux of lactate through the brain		

NSF	GABAB signaling efficacy; synaptic transmission; exocytosis; membrane fusion events; dendritic shaft		
GABARAP	GABA receptor binding; synaptic transmission		
GABARAPL1	GABA receptor binding; autophagy	synaptic transmission/GABA/autophagy	vee
GABARAPL2	GABA receptor binding; autophagy; intra-Golgi vesicle-mediated transport; protein transport		
SCAMP5	SNARE complex component; synaptic vesicle membrane component; positive regulation of calcium ion-dependent exocytosis	synaptic transmission/SNARE complex	vee
STX6	vesicle transport and fusion (t-SNARE family); neurite outgrowth		
CLP1	RNA splicing; mRNA 3'-end processing	transcriptional regulation	circle (yellow)
DENR	contains an SUI1 domain; interects with eIF-2 and directs the ribosome to the proper translation start site		
EIF6	translation initiation factor		
PCF11	degradation of RNA polymerase II-associated nascent RNA and transcriptional termination		
POLR2C	subunit of RNA polymerase II, the polymerase responsible for synthesizing messenger RNA		
R3HDM	poly(A) RNA binding		
ZNF212	gene regulation and development		
ZNF664	zinc finger protein; regulation of transcription		
UBAC1	protein ubiquitination	ubiquitination	parallelogram (pink)
