# Supplementary material for: Community Structure Analysis of Transcriptional Networks Reveals Distinct Molecular Pathways for Early- and Late-Onset Temporal Lobe Epilepsy with Childhood Febrile Seizures
Source: PLoS One. 2015 May 26;10(5):e0128174. doi: 10.1371/journal.pone.0128174 (PMC4444281; doi:10.1371/journal.pone.0128174)
Supplement: S4 Table — Functional description of interactome nodes linked in first and second levels; centered in hubs, high-hubs and VIPs. (PDF) [file pone.0128174.s006.pdf]

Table S4. Late CO interactome network. Functional description of interactome nodes linked in first and second levels - centered in hubs, high-hubs and VIPs.

Protein	PubMed	Biological Process	Shape (border color)
CYCS	initiation of apoptosis	apoptosis	parallelogram
DFFA	substrate for caspase-3 and triggers DNA fragmentation during apoptosis		
PPP2R1A	apoptosis and endoplasmic reticulum (ER) stress associated protein		
PPP2R2B	apoptotic process		
RPS3A	transcription; metastasis; DNA repair; apoptosis		
STK24	MAPK signaling; programmed cells death		
TP53	DNA repair; apoptotic process; cellular stress response		
YWHAQ	apoptotic process		
DFFB	apoptotic process in hippocampus; intracellular signal transduction	apoptosis/hippocampus	parallelogram
GABARAPL2	GABA receptor binding; autophagy; intra-Golgi vesicle-mediated transport; protein transport	autophagy/synaptic transmission/GABA	octagon
ECHS1	hydration of 2-trans-enoyl-coenzyme A (CoA) intermediates	cell processes	circle (pink)
GNL2	GTP catabolic process; ribosome biogenesis		
GOT2	Glutamic-oxaloacetic transaminase; amino acid metabolisms		
LRRC59	cellular component of endoplasmic reticulum membrane		
NAP1L1	DNA replication; positive regulation of cell proliferation		
PAICS	purine nucleobase biosynthetic process		
PCNA	RAD6-dependent DNA repair; cell proliferation; cell cycle		
ROD1	Official Symbol: PTBP3. Regulator of cell differentiation		
TMEM33	transmembrane protein; component of melanosome		
ESR1	estrogen receptor alpha/regulator of energy balance	cell processes/ bioenergetic systems	circle (pink)
TKT	channeling of excess sugar phosphates to glycolysis; energy reserve metabolic process		
HSPE1	heat shock protein which functions as a chaperonin	cell processes/chaperonin	circle (pink)
OLFM2	interactor of Smad3	cell processes/signaling	circle (pink)
YWHAB	signal transduction/14-3-3 protein family		
EPB41	cytoskeletal network	cytoskeleton	circle (green)
MAEA	cytoskeleton organization		
FSCN1	formation of actin-based cellular protrusions; cell migration; motility; adhesion; interactions	cytoskeleton/actin	circle (green)
MTPN	actin-driven cell movements and motility during neuronal development		
PXN	cytoskeleton organization; actin-membrane attachment		
RHPN2	(Rho)-GTPase binding protein; actin cytoskeleton organization		
WAS	actin cytoskeletal organizing		
YWHAZ	14-3-3 protein; cofilin-based regulation of actin filament dynamics; synaptic plasticity		
CACYBP	calcyclin (S100A6) binding neuronal protein; organization of microtubules	cytoskeleton/tubulin	circle (green)
RUVBL1	tubulin association; centrosome		
TACC2	cellular growth and differentiation; centrosomal microtubule stabilization; microtubule organization		
MBNL1	splicing of microtubule-associated protein Tau	cytoskeleton/tubulin-associated	circle (green)
TNF	inflammation	inflammation/cell death	diamond
ANXA2	microtubule-associated tau protein stabilization in cortex neurons; inflammation; S100A10 binding	inflammation/immune function	diamond
MIF	cell-mediated immunity; immunoregulation; inflammation		
AGER	Aliase: RAGE. Receptor for advanced glycation end-products; apoptosis; inflammation	inflammation/neuroglia processes	diamond
S100A11	Ca2(+)-binding protein	ion binding	triangle
KCNH1	potassium voltage-gated channel	ion channel/voltage-gated channel	triangle (red)
S100A6	Calcyclin. Calcium binding protein; nervous system function; upregulation involved in neuronal degeneration; AGER (RAGE) interactor	neuroglia processes	rectangle
S100B	astroglial activation; neuronal synaptic plasticity; axonogenesis; central nervous system development; learning and memory		
TSEN2	tRNA splicing endonuclease subunit involved in brain development	neuronal development	hexagon
BMPR2	brain development; hippocampal neuronal plasticity upon brain injury	neuronal development/plasticity	hexagon
CSK	c-src tyrosine kinase; calcium channel sparklet activity regulation; regulation of NMDAR		
ERBB2	nervous system development; glial cell differentiation; axon guidance		
HPRT1	specification and development of neurons		
ITGAV	cell adhesion; migration; axon guidance		
LCK	Src kinase-family; expressed in neurons of the hippocampus		
PPFIBP1	axon guidance; synapse development		
STK38	Aliase: NDR. Neuron fate specification and circuit formation; dendrite morphogenesis		
TAGLN2	neuron development; estrogen-regulated		
ZIC5	neuronal development		
CBR1	NADPH-dependent oxidoreductase; neurite outgrowth via Stat3 activation	neuronal development/plasticity/dendritic growth	hexagon
CDKN2A	Aliases: p16; INK4A. cell cycle; epigenetic response during nerve regeneration; axonal lenght		
PRKG1	neurite outgrowth; mediators of the nitric oxide/cGMP signaling pathway; spinal synaptic potentiation and pain hypersensitivity		
PTPRZ1	neuritogenesis regulation; neuronal reorganization in MTLE hippocampus		

RANBP1	Ran GTPase system; importin-dependent transport and signaling; cytoskeleton dynamics; neurite outgrowth in mammalian neuron		
SMAD4	dendritic growth and complexity and neuron cell body size		
WDR36	Axon outgrowth		
G3BP2	expression level altered in temperal lobe epilepsy; signal transduction; transport; stress granule formation	neuroprotection	rectangle (red)
P4HB	Aliase: PDI. Act as a chaperone; neuroprotection; subunit of the microsomal triglyceride transfer protein complex		
PHB2	neuronal survival		
PIN1	neuronal differentiation and survival; stress response		
SFN	signal transduction/14-3-3 protein family		
SULT1E1	catalyzes sulfate conjugation of neurotransmitters and hormones; control levels of estrogen receptors; metabolism of cholesterol and neurosteroids	neuroprotection/cholesterol metabolism	rectangle (red)
ABL1	synaptic structure and function regulation; synapse formation in neurons	synaptic transmission	vee
ARRB1	G-protein coupled receptor internalization; positive regulation of Rho protein signal transduction		
DLG4	protein localization to synapse; axon guidance; nervous system development		
GDI1	regulate the GDP-GTP exchange reaction of Rab family members, involved in vesicular trafficking; membrane transport in neurons		
ITGB3	cell adhesion; migration; signaling; modulator of serotonergic systems		
MDM2	ubiquitin-protein ligase activity; zinc ion binding; synaptic transmission		
PEBP1	Aliase: HCNP. Regulation of neurotransmitter levels; stimulates enzymatic activity of choline acetyltransferase in neurons		
S100A1	Ca2+-dependent regulation of synaptic vesicle trafficking; endocytosis; axonal projection; neuroplasticity		
YWHAG	14-3-3 protein; regulation of neuron differentiation; regulation of synaptic plasticity		
PPIB	cyclophilin B. Presynaptic function; membrane tracfficking of GABA receptors	synaptic transmission/GABA	vee
CELF1	Aliase: BRUNOL2. mRNA editing	transcriptional regulation	circle (yellow)
CLP1	RNA splicing; mRNA 3'-end processing		
EIF1	translation initiation factor		
EIF1B	translation initiation factor		
EWSR1	RNA processing and transport		
FTSJ1	RNA processing and modification; hypoexpression associated to intellectual disability, language delay, autistic behaviors, and seizures		
HNRNPH1	mRNA processing; splicing regulator		
HNRNPR	mRNA processing; splicing regulator		
LARP4	cell morphogenesis; mRNA homeostasis		
MLXIP	regulation of genes in response to cellular glucose levels		
NOP56	rRNA processing		
RBBP4	histone acetylation and chromatin assembly; transcriptional repression of E2F-responsive genes		
RPS11	ribosomal protein S11		
RPS9	regulation of cell proliferation; regulation of translation		
SLIRP	steroid receptor RNA activator interaction; regulation of trasncription		
U2AF2	regulation of transcription; mRNA processing		
ARRB2	G-protein coupled receptor binding; ubiquitin protein ligase binding	ubiquitination	parallelogram (pink)
PMPCA	proteolysis		
PSMA1	member of multicatalytic proteinase complex (proteasome), subunit alpha type, 1		
PSMA6	member of multicatalytic proteinase complex (proteasome), subunit alpha type, 6		
PSMB1	member of multicatalytic proteinase complex (proteasome), subunit beta type, 1		
PSMB3	member of multicatalytic proteinase complex (proteasome), subunit beta type, 3		
PSMB5	member of multicatalytic proteinase complex (proteasome), subunit beta type, 5		
PSME3	member of multicatalytic proteinase complex (proteasome), subunit 3		
RMND5B	Aliase: GID2. Degradation of gluconeogenic enzymes via ubiquitin proteasome		
UBE2D2	protein binding; ubiquitination		
UBE2D3	apoptotic process; protein ubiquitination		
UBE2D4	protein ubiquitination		
UBE2E1	cell cycle; ubiquitination		
UBE2E3	protein ubiquitination; regulation of growth		
UBE2W	protein ubiquitination		
USP10	protein ubiquitination; proteolysis		
